# Comparing the efficacy of nanocarriers for cutaneous and follicular delivery of poorly water-soluble molecules: A case study with ciclosporin A^☆^

**DOI:** 10.1016/j.ijpx.2026.100505

**Published:** 2026-02-10

**Authors:** Aditya R. Darade, Maria Lapteva, Yogeshvar N. Kalia

**Affiliations:** a School of Pharmaceutical Sciences, University of Geneva, CMU, 1 rue Michel-Servet, 1211 Geneva, Switzerland; b Institute of Pharmaceutical Sciences Western Switzerland, University of Geneva, Geneva, Switzerland

**Keywords:** Ciclosporin A, Skin, Micelles, Liposomes, Nanoparticles, Nanostructured lipid carriers, Micro−/nanoemulsion

## Abstract

Therapeutic agents approved for the topical treatment of dermatological diseases have diverse physicochemical properties, but they are frequently poorly water-soluble, which makes it a challenge to prepare stable aqueous formulations with good delivery characteristics. Several types of nanocarrier have been reported to facilitate formulation and to enhance cutaneous delivery but there are few direct comparisons of nanocarriers in terms of their ability to deliver a specific molecule to the skin under the same controlled conditions. The present study aimed to address this by developing, optimizing, and comparing different nanocarriers with respect to their ability to deliver ciclosporin A (CsA) to the skin and the hair follicle. Nanoconstructs were categorized as vesicular carriers (micelles and liposomes), emulsion-based systems (microemulsions and nanoemulsions), and nanoparticle systems (e.g. polymeric nanoparticles, solid lipid nanoparticles, nanostructured lipid carriers). Formulations were optimized using a design of experiments approach and were characterized with respect to size, morphology and incorporation efficiency. Cutaneous and follicular delivery experiments were performed using porcine skin. CsA deposition, cutaneous biodistribution, follicular delivery and targeting potential (ratio of delivery to skin with and without pilosebaceous units) were assessed. Nanoemulsions, kinetically stable systems with high thermodynamic activity, showed the highest cutaneous delivery of CsA among the nanosystems tested followed by solid lipid nanoparticles and mPEG-dihexPLA micelles – i.e. three different types of nanocarrier. The results confirmed the pivotal role of thermodynamic activity in determining delivery efficiency of a nanocarrier and its greater importance than other routinely studied morphological parameters such as nanocarrier size: the smallest nanocarriers did not yield the highest delivery.

## Graphical abstract

**Figure:**
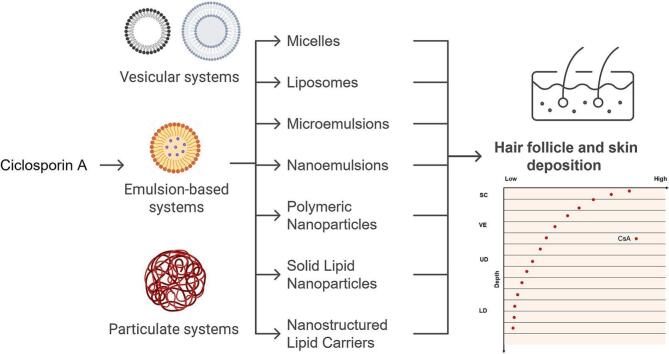

## 1 Introduction

Ciclosporin A (CsA) is a neutral lipophilic cyclic undecapeptide having a molecular weight of 1202 Da (Gupta et al., 1989). It was approved in 1983 (Sandimmune®) for the prevention of organ transplant rejection and an oral formulation (Neoral®) was approved in 1997 for the treatment of psoriasis and rheumatoid arthritis (Rosmarin et al., 2010). CsA freely diffuses into the cytoplasm of T cells, binds to cyclophilin; the resulting complex binds to and inhibits calcineurin, a Ca^2+^/calmodulin-dependent phosphatase, which dephosphorylates nuclear factor of activated T cells (NFAT) allowing it to translocate to the nucleus and so facilitate the transcription of proinflammatory genes coding for IL-2, IL-4, interferon-gamma, transforming growth factor-beta, and up-regulation of the IL-2 receptor. CsA also down-regulates intercellular adhesion molecule-1 on keratinocytes and endothelial cells as well as prevents the recruitment of inflammatory cells into the skin (Gottlieb et al., 1992; Prens et al., 1995; Horrocks et al., 1991).

Currently, CsA is given orally in cases of moderate to severe psoriasis where the disease affects a more significant surface area or plaques are recalcitrant to topical treatments. To-date, and despite its known therapeutic efficacy, there are no approved topical formulations of CsA for cutaneous applications. CsA has a large of volume of distribution and systemic administration results in its “untargeted and nonselective” delivery. Although systemic administration of CsA is clearly justified for indications where the benefits to the patient outweigh the risks of the adverse effects (Sternthal et al., 2008; Marienhagen et al., 2019), long-term systemic CsA exposure is associated with hepatotoxicity, nephrotoxicity, neurotoxicity, hypertension, hypertrichosis, and hyperpigmentation (Marienhagen et al., 2019; Wysocki and Daley, 1987; Wu et al., 2018; Kassianides et al., 1990; Serkova et al., 2004; Sharma et al., n.d.; Abikhair et al., n.d.). In the case of psoriasis where the disease is localized, it would be advantageous to apply CsA directly to the diseased skin; topical delivery would be more targeted and would obviously diminish the risk of off-site effects.

CsA is poorly water-soluble (log P 4.12) (Goyal et al., 2015); its solubility in PBS (pH 7) at room temperature is 5.2 μg/mL (Lallemand et al., 2005). This makes it very difficult to prepare aqueous topical preparations of CsA. Many different nanocarriers – e.g. nanosuspensions (Romero et al., 2016), polymeric nanoparticles (Jain et al., 2011), liposomes (Kumar et al., 2016), nanoemulsion (Musa et al., 2017). microemulsion (Pandey et al., 2020), solid lipid nanoparticle (SLN) and nanostructured lipid carrier (NLC) (Arora et al., 2017) – have been studied using skin from different species, with the aim to improve the cutaneous delivery of CsA. Lapteva et al. developed a micelle-based formulation of CsA and demonstrated its ability to deliver CsA to porcine and human skin at supra-therapeutic concentrations (Lapteva et al., 2014).

The studies described above were performed using a variety of experimental conditions and parameters. There are few systematic studies comparing the abilities of different nanocarriers to deliver poorly water-soluble molecules to the skin under the same controlled conditions. Furthermore, each nanocarrier has its own strengths and weaknesses, posing different challenges and technical hurdles during development and eventual scale up and there is also the question of cost.

Therefore, it was of interest to compare the abilities of different nanocarriers to deliver CsA to the skin under standardized conditions, and to investigate parameters that might affect cutaneous bioavailability and delivery efficiency. Furthermore, determination of the cutaneous biodistribution, and the amounts of CsA present in the pilosebaceous unit (PSU), enabled delivery to the different anatomical layers to be quantified and the ability of the nanocarriers to target specific skin structures to be evaluated. This approach not only allowed the development of the most efficient and robust aqueous “nanocarrier” formulation for cutaneous topical delivery of CsA but also provided insight into differences between the nanocarriers with respect to possible penetration pathways. Of course, the observations made during the study would also have potential applications in the development of nanocarrier-based formulations for the delivery of other poorly water-soluble moleculesto the skin.

The specific objectives of the present study were: (i) to develop and to optimize a series of nanocarrier formulations of CsA using a design of experiments (DoE) approach, (ii) to characterize them with respect to their morphology and CsA incorporation efficiency, (iii) to investigate the cutaneous delivery of CsA from the different nanocarrier formulations, (iv) to assess the cutaneous biodistribution, i.e. CsA delivery as a function of depth, in the skin, (v) to quantify follicular delivery of CsA using a punch biopsy method that enabled the extraction of an intact PSU. In conclusion, the results would enable a systematic comparison of the ability of the nanocarriers to deliver CsA to the skin and the different anatomical regions and to assess the potential for selective/preferential delivery to the PSU.

## 2 Materials and methods

### 2.1 Materials

CsA was kindly provided by Apidel SA (Geneva, Switzerland). mPEG-dihexPLA copolymer (methoxy-poly(ethylene glycol) di-(hexyl-substituted polylactide)) was synthesized in-house as described previously (Trimaille et al., 2006). D-α-Tocopherol polyethylene glycol 1000 succinate (TPGS), formic acid (FA; MS grade), Tween 20, Tween 80, isopentane, and Dulbecco's phosphate-buffered saline (DPBS), Resomer® RG 503H (PLGA; MW: 24–38 kDa), polyvinylpyrrolidone (PVP; MW: 360 kDa), castor oil, Kolliphor® EL, oleic acid, palmitic acid, myristic acid, chloroform were purchased from Sigma-Aldrich (Buchs, Switzerland). Lipoid S100 was kindly provided as a gift by Lipoid GmbH (Ludwigshafen, Germany). Stearic acid was purchased from Hänseler AG (Herisau, Switzerland). Bovine serum albumin (BSA), polyethylene glycol 400 were purchased from Axon Lab (Baden-Dättwil, Switzerland). Acetone (analytical grade), lauric acid, and Nile Red were obtained from Acros Organics (Geel, Belgium). Glycerol monostearate (GMS) was procured from BASF (Ludwigshafen, Germany). Transcutol® P, Labrafac™ WL 1349, Labrasol® were procured from Gattefosse (Saint Priest, France). Miglyol® 840 was obtained from Cremer Oleo GmbH (Hamburg, Germany). Methanol and acetonitrile (LC-MS grade), dichloromethane (DCM) were purchased from Fisher Scientific (Reinach, Switzerland). PTFE membrane filters (0.22 μm), Amicon Ultra 0.5 mL (5 kDa) filtration units were purchased from VWR (Nyon, Switzerland). Ultrapure water (Millipore Milli-Q Gard 1 Purification Pack resistivity >18 MΩ.cm; Zug, Switzerland) was used for formulation development and analysis. All other chemicals were at least of analytical grade.

### 2.2 Analytical methods

CsA was quantified using a Waters Acquity I-class UPLC® system equipped with a Xevo® TQ-S micro tandem quadrupole detector. Gradient separation was performed using an Acquity UPLC® BEH C18 column (2.1 × 50 mm; 1.7 μm) in tandem with an Acquity UPLC® C18 VanGuard pre-column (2.1 × 5 mm, 1.7 μm), which was maintained at 25 °C. The mobile phase consisted of a mixture of methanol (0.1% FA) and water (0.1% FA), 65:35 *v*/v at *t* = 0 min, which changed to 95:5 v/v at *t* = 0.3 min, this was maintained until *t* = 2 min followed by a return to the initial composition at *t* = 2.1 min. The flow rate and injection volume were 0.1 mL/min and 5 μL, respectively. The CsA peak was observed at 3.2 min, and the total run time was 5.0 min. Mass spectrometric detection was performed with electrospray ionization in positive ion mode using multiple reaction monitoring (MRM). The detection settings for CsA are presented in Table 1. The limits of detection (LOD) and quantification (LOQ) were 1.61 and 4.90 ng/mL, respectively. The UHPLC-MS/MS method was validated as per ICH guidelines (complete details are provided in the **Supplementary Data, Section** 1 – **Fig. S1** and **Table S1**).

**Table 1:** MS/MS Settings for the detection of CsA.

	CsA
Nature of precursor ion	Hydrogen adduct [M + H]^+^
Precursor ion (*m*/*z*)	1202.74
Product ion (m/z)	224.27
Collision energy (V)	52
Cone voltage (V)	76
Capillary voltage (kV)	4
Desolvation temperature (°C)	300
Desolvation gas flow (L/h)	800
Cone gas flow (L/h)	1
LM resolution 1	9.7
HM resolution 1	15.1
Ion energy 1 (V)	0.6
LM resolution 2	9.8
HM resolution 2	15
Ion energy 2 (V)	0.6

### 2.3 Preparation of nanoformulations

The different aqueous nanoformulations of CsA were prepared with a target drug content of 2 mg_CsA_/mL_formulation_ (i.e. 0.2% *w*/*v*). These included (i) vesicular nanocarriers – micelles and liposomes, (ii) particulate nanocarriers – with examples of both polymeric and lipid nanocarriers (solid lipid nanoparticles (SLN) and nanostructured lipid carriers (NLC), and (iii) emulsion-based nanosystems – microemulsions and nanoemulsions. While developing the nanoformulations, the following baseline criteria were considered to enable a systematic comparison: (i) nanocarrier size should be in the same range, (ii) the excipient(s) should be used in the minimum amounts required to avoid excipient-related effects, e.g. using more than the required concentrations of PLGA in preparing polymeric nanoparticles may hinder drug release, and (iii) nanoformulations should be physicochemically stable for at least 4 weeks at 4 °C.

Apart from the mPEG-dihexPLA micelles, and emulsion-based nanosystems, all other nanoformulations were optimized by the design of experiments (DoE) approach: (i) mPEG-dihexPLA micelles of CsA were prepared as previously described by Lapteva et al. (Lapteva et al., 2014) and, (ii) in the case of emulsion-based systems, CsA solubility determination in emulsion components and the modified water titration method, which screened for emulsion regions, provided relevant information for optimization of the latter without requiring use of DoE.

Optimization of robust formulations with the desired properties was achieved using Stat Ease-Design Expert® 13 software. Response surface methodology (RSM), based on a central composite design (CCD), was used to optimize the nanoformulations. CCD is a factorial or fractional factorial design that consists of set experiments augmented with center point experiments and additional point experiments outside the set experimental domain for each factor (also called star points) that allows the estimation of the response surface curvature (Ait-Amir et al., 2015). Fractional factorial design CCD is used in the optimization of the formulation with the desired properties, but with fewer experiments than required for the full factorial design approach. For four factors having three levels of each factor, a total of 3^4^ (81) experiments is required for full factorial design, whereas for the same case, a total of 28 experiments would be needed for CCD with four center point experiments. This enables more information to be obtained in fewer experiments and saves time, effort and resources. The number of experiments (N) in CCD is determined using Eq. 1 where k is the number of factors and N_0_ are the central points:

(1) $$N=2^{k}+2k+N_{0}$$

CsA nanoformulation influencing factors and the response used in DoE are presented in Table 2. NLCs were developed by adding liquid lipid in the optimized SLN formula with minimal changes.

**Table 2:** Tested factors and response in CCD approach for the development of CsA formulations.

Formulation	Factors of influence	Factor Range	Measured response
Micelles	[CsA]	0.1–8.24 (mg/mL)	Entrapment efficiency
	[TPGS]	2.93–17.07 (mg/mL)	
Liposomes	[CsA]	0.38–4.62 (mg/mL)	
	[Lipoid S100]	10–94.5 (mg/mL)	
Polymeric nanoparticles	Drug concentration	1–4 (mg/mL)	
	[Resomer® RG 503H]	20–95 (mg/mL)	
	[Tween 20]	0–3%	
	[PVP]	1–5%	
Solid lipid nanoparticles	[Glyceryl monostearate]	6.48–48.52 (mg/mL)	
	[Lipoid S100]	0.1–0.64%	
	[Tween 20]	0.1–3.99%	

The entrapment efficiency of the nanoformulations was determined by ultra-filtration using Amicon Ultra-0.5 mL centrifugal filters with pore size cut off 3 kDa (nominal pore size 0.3 nm) (**Supplementary Data, Section 5 – Table S5**). The pH of all nanoformulations was neutral.

#### 2.3.1 Micelle formulations

The mPEG-dihexPLA based micelles of CsA were prepared by solvent evaporation as described previously (Lapteva et al., 2014). Trimaille et al. (Trimaille et al., 2006) developed a methoxy-poly(ethylene glycol)-poly(hexylsubstituted lactides) (mPEG-dihexPLA) diblock copolymer: the methoxy-poly(ethylene glycol) chain constituted the hydrophilic region, whereas the hexyl substituted polylactide constituted the lipophilic part of the surfactant. The presence of hexyl groups resulted in an increased lipophilicity of the core facilitating the incorporation of highly lipophilic drugs and decreased the critical micelle concentration to 1.2 μM. The formulations were reproducible with similar physicochemical characteristics. Briefly, known amounts of surfactant and CsA were dissolved in 2 mL of acetone to obtain a clear solution. This solution was added slowly to 4 mL water under sonication (Branson Digital Sonifier S—450D). Acetone was then slowly removed by using a rotary evaporator (Büchi RE 121 Rotavapor). The final volume was made up with water in a volumetric flask to obtain the micelle formulation with CsA and mPEG-dihexPLA concentrations of 2 mg/mL and 5 mg/mL, respectively. After equilibration overnight, the micelle solution was centrifuged at 10,000 rpm for 15 min (Eppendorf Centrifuge 5804) to remove excess CsA, and the supernatant was carefully collected.

D-α-Tocopherol polyethylene glycol 1000 succinate (TPGS) is a nonionic amphiphilic surfactant synthesized by the esterification of vitamin E succinate. It has been categorized as a safe excipient by the US Food and Drug Administration (FDA) and the European Medicine Agency (EMA) (Rathod et al., 2021). TPGS spontaneously forms kinetically stable core–shell micelles (diameter 12–15 nm) in water above its CMC (20 μM) (Rathod et al., 2021). TPGS based micelles were optimized as described above by using CCD. Thirteen randomized experiments were performed with CsA and TPGS concentrations as the factors and entrapment efficiency as the response. The data were entered into Stat Ease-Design Expert® 13 to obtain the formulation with the target properties. A micelle formulation with CsA and TPGS concentrations of 2 mg/mL and 10 mg/mL, respectively, was optimized.

#### 2.3.2 Liposome formulation

Soybean phosphatidylcholine (Lipoid S100; PC >96%) has been extensively used in drug delivery to encapsulate drugs in lipid bilayer vesicles to improve their efficacy. Lipoid S100 based liposomes of CsA were prepared by thin-film hydration (Gottlieb et al., 1992; Prens et al., 1995). Thirteen randomized experiments were performed with CsA and lipid concentrations as the factors and entrapment efficiency as the response in the CCD. Known amounts of lipid and drug were dissolved in chloroform. The chloroform was removed by using a rotary evaporator (Büchi RE 121 Rotavapor) at 40 °C under reduced pressure to obtain a uniform thin film. The film was hydrated using MilliQ water to obtain large multilamellar vesicles. These vesicles were subjected to probe ultrasonication (Branson Sonifier® SFX 250) at 30% amplitude for 3 min to obtain small unilamellar vesicles. After equilibration overnight, the liposome solution was centrifuged at 10,000 rpm for 15 min (Eppendorf Centrifuge 5804) to remove excess CsA, and the supernatant was carefully collected. The liposome formulation with CsA and Lipoid S100 concentrations of 2 mg/mL and 40 mg/mL, respectively, was optimized.

#### 2.3.3 Polymeric nanoparticles (NP)

Poly(lactic-*co*-glycolic acid) (PLGA) is a biodegradable and biocompatible polymer with a wide range of degradation times that can be modified by its molecular weight and copolymer ratio. PLGA micro- and nanoparticles have been widely studied as delivery vehicles for low molecular weight drugs, peptides and proteins and other macromolecules such as DNA and RNA (Operti et al., 2021). Resomer® RG 503H (PLGA; MW: 24–38 kDa) based NP of CsA were prepared by the emulsification-solvent evaporation method (Horrocks et al., 1991; Mason, 1990). Since the approval of the first poly(lactic-*co*-glycolic acid) (PLGA) microsphere based formulation, Decapeptyl® SR in 1986, PLGA has been widely used in drug delivery due to its excellent biocompatibility, and tunable release properties. It has been approved by both the US FDA and the EMA (Operti et al., 2021). It is usually synthesized by the ring-open co*-*polymerization of lactide and glycolide. The drug release properties of PLGA depend on the lactide (lipophilic):glycolide (hydrophilic) ratio, monomer order, and molecular weight (Gentile et al., 2014; Rampaka et al., 2021).

Twenty randomized experiments were performed with CsA, Resomer® RG 503H, Tween 20, and PVP concentrations being factors and the entrapment efficiency as the response in the CCD. Briefly, known amounts of CsA and Resomer® RG 503H were dissolved in DCM. Tween 20 (stabilizer) and PVP (thickener) were dissolved in water. The two phases were mixed and emulsified using an IKA® T25 digital ULTRA-TURRAX® at 3000 RPM for 2 min followed by probe ultrasonication (Branson Sonifier® SFX 250) at 40% amplitude for 3 min in an ice bath to avoid the evaporation of DCM due to the heat generated. DCM was then removed by using a rotary evaporator (Büchi RE 121 Rotavapor). The final volume was made up with water in a volumetric flask to obtain NP with CsA and Resomer® RG 503H concentrations of 2 mg/mL and 40 mg/mL, respectively.

#### 2.3.4 Solid lipid nanoparticle (SLN) and nanostructured lipid carrier (NLC) formulations

SLN have emerged as a potential delivery system with many of the advantages of fat emulsions, polymeric nanoparticles, and liposomes for drug delivery. SLN can be used to improve solubility and loading of hydrophobic drug molecules and enhance stability due to the encapsulation of drugs (Rampaka et al., 2021). SLN were prepared by the hot homogenization method (Wu et al., 2018; Kassianides et al., 1990). Fourteen randomized experiments were performed. Glyceryl monostearate (GMS), Lipoid S100, and Tween 20 concentrations were chosen as factors and the entrapment efficiency as the response in the CCD. The solubility of CsA was determined in different solid lipids. Briefly, 100 mg of CsA was placed in a test tube, the solid lipid was added in increments of 100 mg, and the test tube was heated in a controlled temperature oil bath kept at 80 °C. The minimal amount of lipid required to solubilize the CsA in a molten state was determined (Joshi and Patravale, 2008). CsA showed similar solubility in the tested solid lipids. Sanna et al. (Sanna et al., 2010) reported an increase in drug release with increasing chain length of solid lipids; therefore, GMS was selected. It was also considered to be less prone to pH changes during formulation preparation, storage and drug delivery compared to the other tested conventional fatty acids. CsA was dissolved in molten GMS and Lipoid S100 mixture. Tween 20 was added to water and heated to the same temperature. Both phases were mixed and emulsified by using a probe ultrasonicator (Branson Sonifier® SFX 250) at 40% amplitude for 3 min to obtain a hot nanoemulsion, which was quickly cooled on an ice bath to obtain the SLN. The volume lost due to the evaporation by sonicating the hot emulsion was made up with water after equilibration overnight.

For NLC, medium-chain triglyceride (MCT; Labrafac™ WL1349) was used as the liquid lipid while the other components and the process were similar to that for preparation of the SLN.

#### 2.3.5 Microemulsion (ME) and nanoemulsion (NE) formulations

ME and NE are emulsions with globules in the “nano-” size range. The key difference is that ME are thermodynamically stable systems that are formed spontaneously, whereas NE are thermodynamically unstable/kinetically stable systems that usually require external energy for their formation (Callender et al., 2017). Interfacial tension of ME is ultralow and usually ranges from 0.001 to 0.1 mN/m whereas that of NE is usually >1 mN/m (Callender et al., 2017; Phan et al., 2011; Kumar and Mandal, 2018). CsA solubility in various surfactants and oils was determined. An excess of the drug was added individually to oil, and surfactant (5 mL each) in screw-capped tubes. After 24 h, each sample was centrifuged and the clear supernatant layer was diluted with methanol and analyzed by UHPLC-MS/MS. The ME region of the selected surfactant-oil system was determined by the modified water titration method in 48 well plates. Approximately 100 μL of mixtures of oil and surfactant in different proportions were placed in the microwells and the water phase was added to the wells to screen the ME region. Ternary phase diagrams with components being oil phase, water phase and surfactant mixture having four different ratios of surfactants were generated to select the suitable surfactant mixture and final ME composition. Once the suitable composition was selected, the drug was dissolved in the oil-surfactant mixture, and the water phase was added to obtain the clear ME.

For NE, a similar oil-surfactant system was used to avoid variation in delivery experiments due to the use of different excipients. A suitable composition in the emulsion region (biphasic region) was screened and selected. The globule size was reduced by using a probe ultrasonicator (Branson Sonifier® SFX 250) at 40% amplitude for 5 min in an ice bath to obtain a translucent NE.

### 2.4 Characterization of nanoformulations

*Size determination:* The hydrodynamic diameter (Z_av_), polydispersity index (P.D.I.), and volume-weighted and number weighted diameters (d_v_ and d_n_, respectively) of the nano-formulations were measured using dynamic light scattering (DLS) with a Zetasizer ZS (Malvern Instruments Ltd.; Malvern, UK). Measurements were performed at an angle of 90° and a temperature of 25 °C. All values were obtained after 3 runs of 10 measurements.

*Morphology observation:* The morphology of the nanoformulations was characterized with transmission electron microscopy (TEM) (FEI Tecnai G2 Sphera, Eindhoven, Netherlands) using the negative staining method. Briefly, 5 μL of the nanoformulation was dropped onto an ionized carbon-coated copper grid (0.3 Torr, 400 V for 20 s). The grid was then placed for 1 s in a 100 μL drop of a saturated uranyl acetate aqueous solution and then in a second 100 μL drop for 30 s. The excess staining solution was removed, and the grid was dried at room temperature before the measurement.

*Determination of CsA Content in the nanoformulations:* CsA loaded into the nanoformulations was quantified by UHPLC-MS/MS. The drug content, drug loading, and entrapment efficiency were calculated using Eqs. (2), (3), (4):

(2) $$\mathrm{CsA}\;\mathrm{content}\;\left(\frac{\mathrm{mg}}{\mathrm{mL}}\right)=\frac{\mathrm{CsA}\;\mathrm{in the formulation}\;\left(\mathrm{mg}\right)}{\mathrm{Volume of the formulation}\;\left(\mathrm{mL}\right)}$$

(3) $$\mathrm{CsA}\;\mathrm{loading}\;\left(\frac{\mathrm{mg}\;\mathrm{of}\;\mathrm{CsA}}{g\;\mathrm{of polymer}}\right)=\frac{\mathrm{CsA}\;\mathrm{in the formulation}\;\left(\mathrm{mg}/\mathrm{mL}\right)}{\mathrm{Polymer in the formulation}\;\left(g/\mathrm{mL}\right)}\;$$

(4) $$\mathrm{Entrapment efficiency}\;\left(\%\right)=\frac{\mathrm{CsA}\;\mathrm{entrapped in nanocarrier}\;\left(\mathrm{mg}\right)}{\mathrm{CsA}\;\mathrm{added}\;\left(\mathrm{mg}\right)}\times 100$$

### 2.5 Biopharmaceutical evaluation of nanocarrier systems

#### 2.5.1 Skin preparation

Porcine ears were purchased from a local abattoir (CARRE; Rolle, Switzerland) shortly after sacrifice. After washing under running cold water, skin samples with a thickness of ∼0.8 mm were carefully harvested from the outer region of the ear using a Zimmer air dermatome (Münsingen, Switzerland). Hair was removed from the skin surface using clippers. Discs corresponding to the application area were punched out (Berg & Schmid HK 500; Urdorf, Switzerland). Skin samples were frozen at −20 °C and stored for a maximum period of 3 months. Before the experiment, skin samples were thawed at room temperature and placed for 15 min in 0.9% saline solution for rehydration.

#### 2.5.2 Experimental protocol for cutaneous and follicular delivery studies

A series of experiments were performed using standard two-compartment vertical diffusion cells (Milian SA; Meyrin, Switzerland) with a cross-sectional area of 2 cm^2^, to determine the cutaneous deposition and transdermal permeation of CsA from the different nanoformulations as a function of application time. Nanoformulations or control (2 mg/mL CsA solution in propylene glycol) were placed on the skin surface to the donor compartment; the amount of each formulation ensured that the same “dose” of CsA was applied (500 μg/cm^2^ CsA). The receiver compartment contained 10 mL DPBS pH 7.4 containing 1% BSA to maintain sink conditions. The receiver compartment was maintained at 32–34 °C. Aliquots (1 mL) were withdrawn from the receiver compartment at 1, 4, 8, and 12 h and replaced with an equivalent volume of fresh media. Samples were diluted in acetonitrile to precipitate BSA. After centrifugation at 10,000 rpm for 15 min, the permeation samples were analyzed by UHPLC-MS/MS.

Upon completion of the experiments, the excess formulation was removed from the skin surface using the validated wash method (**Supplementary Data, Section** 4 **– Table S4**). Using a punch, the skin sample was split into two parts – a 0.785 cm^2^ inner disc and the remaining outer ring with an area of 1.215 cm^2^. The outer ring was subsequently cut into small pieces and CsA deposited in the skin was extracted by soaking the pieces in 2 mL of methanol for 4 h with continuous stirring at room temperature. The extraction procedure was validated (**Supplementary Data, Section** 3 **– Table S3**). The extraction samples (at 4, 8, and 12 h) were centrifuged at 10,000 rpm for 15 min, diluted, and filtered through a 0.22 μm PTFE filter before UHPLC-MS/MS analysis (**Supplementary Data, Section** 2 **– Table S2**).

The 0.785 cm^2^ discs were used to determine the CsA biodistribution as a function of depth in the skin. The skin discs were snap-frozen in isopentane cooled by liquid nitrogen. For this, the skin samples were fixed with O.C.T. on a circular piece of cork and a plastic o-ring placed around the skin discs to avoid tissue compression and to ensure a flat frozen sample. This ensured the integrity of the thickness of different regions of the skin. The skin discs were then cryosectioned (Thermo Scientific CryoStar™ NX70; Reinach, Switzerland) to obtain 40 μm thick sections starting from the stratum corneum down to a depth of 400 μm. These lamellae enabled the amounts of CsA to be determined as a function of position in the skin, encompassing the stratum corneum, epidermis, and upper dermis, respectively. Each lamella and the remaining dermis were individually extracted in 250 μL methanol for 4 h and CsA content was quantified by UHPLC-MS/MS.

CsA delivery to the hair follicle from different nanoformulations was studied by using a previously described method (Lapteva et al., 2015; Kandekar et al., 2018; Dahmana et al., 2021), which enabled harvesting of the intact pilosebaceous unit (PSU) with a punch (**Supplementary Data, Section 9 – Fig. S4**). Data were compared to control biopsies where no PSU was present. After completion of the delivery experiments, the skin surface was cleaned using the protocol described above. The PSU or PSU-free control biopsy was harvested using a 1 mm diameter punch (Berg & Schmid HK 500; Urdorf, Switzerland). The harvested biopsy samples were inspected using a Leica S6D microscope (Leica Microsystems (Schweiz) AG; Heerbrugg, Switzerland) to confirm the presence of an entire PSU or to establish that the skin sample was a PSU-free region. CsA deposited in each biopsy sample was extracted for 4 h using 100 μL of methanol at room temperature on a shaker bath at 150 rpm in an Eppendorf tube. The samples were centrifuged at 10,000 rpm for 10 min and CsA was quantified using UHPLC-MS/MS. Delivery efficiency and follicle targeting ability (FTA) was calculated using Eqs. (5), (6):

(5) $$\mathrm{Follicle targeting ability}=\frac{\mathrm{CsA}\;\mathrm{delivered to}\;\mathrm{PSU}\;\mathrm{biopsy}\;}{\mathrm{CsA}\;\mathrm{delivered to control biopsy}}$$

(6) $$\mathrm{Delivery efficiency}\;\left(\%\right)=\frac{\mathrm{CsA}\;\mathrm{deposition}\;\mathrm{at}\;12\;h\;\left(\mathrm{\mu g}/{\mathrm{cm}}^{2}\right)}{\mathrm{CsA}\;\mathrm{applied to the skin}\;\left(\mathrm{\mu g}/{\mathrm{cm}}^{2}\right)}\times 100$$

### 2.6 Statistical analysis

Data were expressed as the mean ± SD. Outliers determined using the Grubbs test were discarded. Results were evaluated statistically using analysis of variance (ANOVA) one-way followed by the Tukey test for multiple comparisons or by Student's *t-*test. The level of significance was fixed at α = 0.05.

## 3 Results and discussion

### 3.1 Formulation development and characterization

**Fig. 1:**
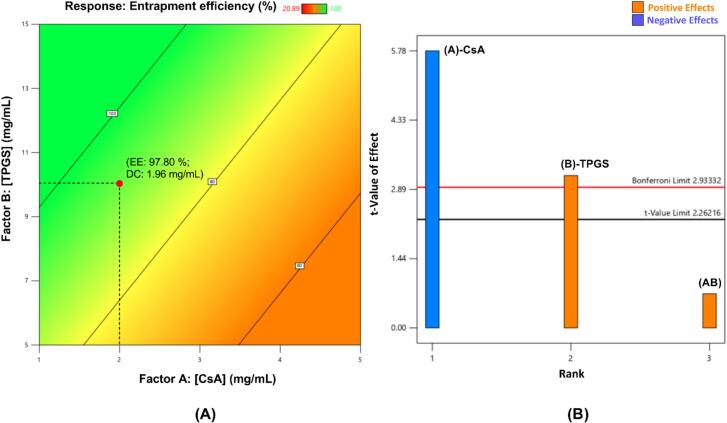
(A) Contour plot for CsA entrapment efficiency of TPGS micelles. Abbreviations (for selected formulation in red dot) given as EE entrapment efficiency, DC drug content, both being experimental values; (B) Pareto chart for determining factor significance in TPGS micelles experimental design. (For interpretation of the references to colour in this figure legend, the reader is referred to the web version of this article.)

**Fig. 2:**
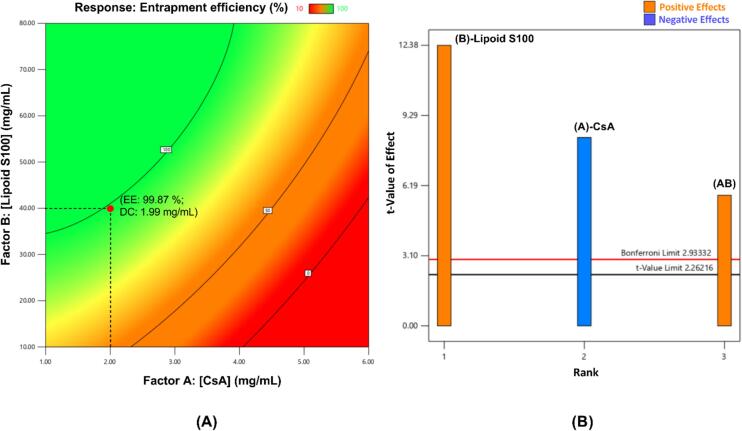
(A) Contour plot for CsA entrapment efficiency of liposomes. Abbreviations (for selected formulation in red dot) given as EE entrapment efficiency, DC drug content, both being experimental values; (B) Pareto chart for determining factor significance in liposomes experimental design. (For interpretation of the references to colour in this figure legend, the reader is referred to the web version of this article.)

**Fig. 3:**
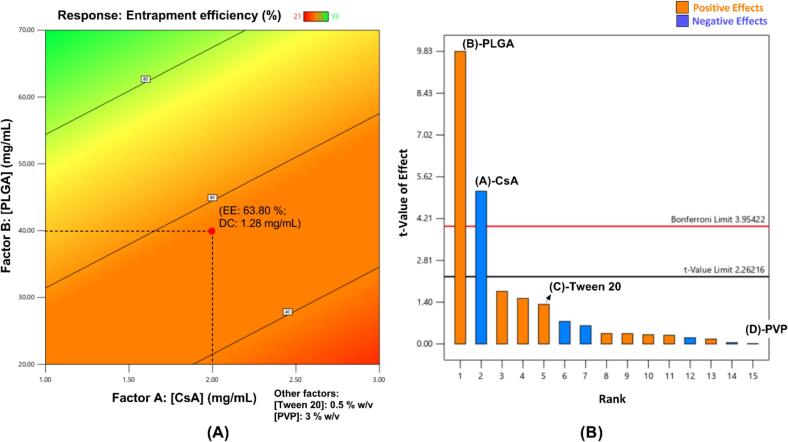
(A) Contour plot CsA for entrapment efficiency of Resomer® RG 503H (PLGA) nanoparticles. Abbreviations (for selected formulation in red dot) given as EE entrapment efficiency, DC drug content, both being experimental values; (B) Pareto chart for determining factor significance in Resomer® RG 503H (PLGA) nanoparticle experimental design. (For interpretation of the references to colour in this figure legend, the reader is referred to the web version of this article.)

**Fig. 4:**
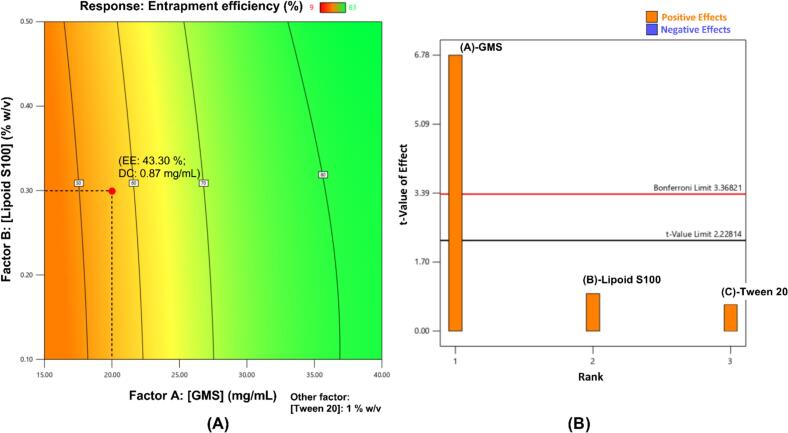
(A) Contour plot for CsA entrapment efficiency of SLN. Abbreviations (for selected formulation) given as EE entrapment efficiency, DC drug content, both being experimental values; (B) Pareto chart for determining factor significance in SLN experimental design.

For all the formulations optimized by DoE, a contour plot was used to select the formulation with the target properties (Fig. 1, Fig. 2, Fig. 3, Fig. 4). The desired response (in this case, entrapment efficiency) was selected by choosing suitable values of the factors utilized for the experimental design. Entrapment efficiency close to 100% is shown by a green colored region whereas the minimum entrapment efficiency region is indicated by the red region. The selected formulations are shown as red dots in the respective contour plots.

#### 3.1.1 Micelle formulation

The mPEG-dihexPLA micelles of CsA were reproduced as reported by Lapteva et al. (Lapteva et al., 2014). The target CsA loading was 400 mg_CsA_/g_polymer_ in the case of mPEG-dihexPLA micelles (Table 3).

**Table 3:** Characterization of different nanocarrier formulations with respect to drug content and size.

Formulation	Excipients (mg/mL)				Target CsA loading (mg_CsA_/g_excip’t_)	CsA assay			Size				Zeta potential (mV)
						CsA loading ± SD (mg_CSA_/g_excipient_)	CsA content ± SD (mg_CSA_/mL_Form_)	Entrapment efficiency ± SD (%)	Z_av_ (nm)	P.D.I.	d_v_ (nm)	d_n_ (nm)	
Vesicular nanosystems	**mPEG-dihex PLA**		**TPGS**	**Lipoid S100**									
mPEG-dihex PLA micelles	5		–	–	400	374.4 ± 22	1.87 ± 0.11	93.60 ± 5.50	32.0	0.257	20.40	15.64	−6.3
TPGS micelles	–		10	–	200	195.6 ± 9.0	1.96 ± 0.09	97.80 ± 4.50	11.5	0.046	10.24	8.83	−4.9
Liposomes	–		–	40	50	49.94 ± 1.20	1.99 ± 0.048	99.87 ± 2.40	87.4	0.109	74.88	56.14	−0.43

Particle-based nanosystems	**Resomer® RG 503H (PLGA; MW: 24–38 kDa)**		**Tween 20** **(% w/v)**	**PVP** **(% w/v)**									
Polymeric nanoparticles	40		0.5	3	50	31.90 ± 2.25	1.28 ± 0.09	63.80 ± 4.50	102.7	0.582	32.15	24.43	−26.3

	**GMS**	**MCT**	**Lipoid S100** **(% w/v)**	**Tween 20 (% *w*/*v*)**									
Solid lipid nanoparticles	20	–	0.3	1	100	43.30 ± 6.30	0.87 ± 0.13	43.30 ± 6.30	272.2	0.248	427.70	150.80	−18.5
Nanostructured lipid carriers	15	10	0.3	1.5	80	46.64 ± 2.32	1.17 ± 0.06	58.30 ± 2.90	147.9	0.104	151.90	108.40	−19.9

Emulsion-based nanosystems	**Oleic acid** **(% v/v)**		**Labrasol: Transcutol P (% v/v)**	**Water** **(% *v*/v)**	**Target drug content (mg**_**CSA**_**/mL**_**Formulation**_**)**								
Microemulsion	8		50	42	2	–	1.86 ± 0.06	93.10 ± 3.10	105.8	0.133	101.10	77.03	−0.44
Nanoemulsion	2		8	90	2	–	1.84 ± 0.16	91.90 ± 7.80	202.8	0.173	215.00	161.60	−4.20

In the case of TPGS micelles, the contour plot was used to select the final optimized formulation (Fig. 1A). A normal plot of residuals (**Supplementary Data, Section 7 – Fig. S2**) was used to check whether or not the data residuals were normally distributed and showed that the data were indeed normally distributed and were further analyzed by ANOVA. The *p*-value (*p* = 0.0002) suggested the experimental design was significant and the tested factors had significant effects on the response. This was also confirmed by the Pareto chart (Fig. 1B), which shows whether the tested factors have a significant effect on the response. In the Pareto chart, the ‘Bonferroni limit’ is the threshold above which the factors and/or their interaction effects are highly significant. The factors above the t-limit but below the Bonferroni limit may possibly be significant, whereas the factors below the threshold of the t-limit are considered to be insignificant (Hu et al., 2016). Both factors, i.e. the CsA and TPGS concentrations, had significant effects on entrapment efficiency. CsA had a negative effect (showed in blue) with increasing concentration while TPGS had a positive effect (shown in orange). The third rank/effect (AB) in Fig. 1B corresponds to the interaction between the two factors and this was below the t-limit and hence insignificant. Moreover, the small difference (< 0.2) between Adjusted R^2^ (0.7910) and Predicted R^2^ (0.6579), indicated the relevance of the developed experimental design. If the experimental design is significant, and there is a good agreement between adjusted and predicted R^2^ (difference < 0.2), then the design can be considered to provide good response predictions. The predicted CsA entrapment efficiency of TPGS micelles was 93.14%.

The drug loadings, drug contents, incorporation efficiencies, and zeta potential obtained for TPGS and mPEG-dihexPLA micelles are given in Table 3. The sizes of the mPEG-dihexPLA and TPGS micelles were 32.0 nm and 11.5 nm, respectively. TPGS being a smaller monomer unit has been found to produce smaller micelles. The TPGS micelles had a CsA entrapment efficiency of 97.80% compared to mPEG-dihexPLA micelles, where the entrapment efficiency was 93.60% (Darade et al., 2023; Gou et al., 2022; Quartier et al., 2021a).

#### 3.1.2 Liposome formulation

The contour plot used to select the Lipoid S100 liposomal formulation with the desired properties is show in Fig. 2A. The normal plot of residuals showed that data were normally distributed and were further analyzed by ANOVA. The *p*-value (*p* < 0.0001) suggested the experimental design was significant and the tested factors had significant effects on the response. This was also confirmed by the Pareto chart (Fig. 2B). As for the micelle formulations, both CsA and Lipoid S100 concentrations had significant effects on entrapment efficiency: CsA had a negative effect (shown in blue) with increasing concentration while Lipoid S100 had a positive effect (shown in orange). The experimental design found an interaction between the two factors (AB, Fig. 2B) which had a significant positive effect on the response. Moreover, the small difference (< 0.2) between Adjusted R^2^ (0.9429) and Predicted R^2^ (0.7903), indicated the relevance of the developed experimental design. The predicted CsA entrapment efficiency of Lipoid S100 liposomes was 99.62%.

The drug loading, drug content, incorporation efficiency and zeta potential for the liposome formulation are given in Table 3. Particle size and entrapment efficiency of Lipoid S100 liposomes were found to be 87.4 nm and 99.87% respectively.

#### 3.1.3 Polymeric nanoparticles

The contour plot for Resomer® RG 503H (PLGA; MW: 24–38 kDa) nanoparticles was used to select the formulation with the desired properties (Fig. 3A). The normal plot of residuals showed that data were normally distributed and were further analyzed by ANOVA. The *p*-value (*p* < 0.0001) suggested the experimental design was significant and the tested factors had significant effects on the response. This was also confirmed by the Pareto chart (Fig. 3B). Both CsA and PLGA concentrations had significant effects on entrapment efficiency: CsA had a negative effect (shown in blue) with increasing concentration while PLGA had a positive effect (shown in orange). The remaining factors – Tween 20 (surfactant) and PVP (thickener) concentrations were found to be insignificant along with various possible interactions. Therefore, the results are shown as a contour plot with fixed values for Tween 20 and PVP concentrations, rather than as a 3D surface plot. Moreover, the small difference (< 0.2) between Adjusted R^2^ (0.9108) and Predicted R^2^ (0.8741), indicated the relevance of the developed experimental design. The predicted CsA entrapment efficiency of PLGA nanoparticles was 57.41%. The formulations with higher entrapment efficiency were not physically stable – probably due to the high PLGA content inducing physical instability through aggregation – and hence were not selected. The concentrations of Tween 20 (surfactant) and polyvinylpyrrolidone (PVP; thickening agent) were optimized and selected as 0.5% and 3%, respectively.

The drug loading, drug content, incorporation efficiency, and zeta potential for the PLGA NP formulation are given in Table 3. The experimentally determined particle size and entrapment efficiency of Resomer® RG 503H (PLGA; MW: 24–38 kDa) nanoparticles were found to be 102.7 nm and 63.8% respectively. The slightly negative zeta potential might contribute to the additional stability of the formulation.

#### 3.1.4 Lipid nanocarriers

CsA was not taken as a factor in the CCD as its solubility had already been determined in the components (Table 4) and while designing the experiments, concentrations of excipients were selected accordingly so as to achieve the desired CsA content (2 mg/mL).

**Table 4:** Solubility of CsA in solid lipids.

Solid lipid	CsA solubility (mg/g lipid)
Lauric acid (C12)	100.90 ± 7.40
Myristic acid (C14)	100.70 ± 11.20
Palmitic acid (C16)	100.40 ± 9.10
Stearic acid (C18)	113.40 ± 3.40
Glyceryl monostearate (C18)	101.20 ± 5.70

The contour plot for GMS solid lipid nanoparticles (SLN) was used to select the formulation with the desired properties (Fig. 4A). The normal plot of residuals showed that data were normally distributed and were further analyzed by ANOVA. The *p*-value (*p* < 0.0001) suggested that the experimental design was relevant, and that the tested factors had significant effects on the response. This was also confirmed by the Pareto chart (Fig. 4B). GMS content had a significant effect on entrapment efficiency. Remaining factors, Lipoid S100 (lipophilic surfactant) and Tween 20 (hydrophilic surfactant) concentrations were found to be insignificant. Therefore, the results are shown as contour plot with fixed value for Tween 20 rather than showing in a 3D surface plot. Moreover, the small difference (< 0.2) between Adjusted R^2^ (0.9598) and Predicted R^2^ (0.8278), confirmed the relevance of the developed experimental design. The concentration of Tween 20 was optimized and fixed at 1%. The predicted CsA entrapment efficiency of SLNs was 56.42%. The formulations with higher entrapment efficiency were not physically stable probably due to high GMS content and hence were not selected.

A medium-chain triglyceride (MCT; Labrafac™ WL1349) was used as a liquid lipid component for preparation of the nanostructured lipid carriers (NLC) with slight modifications to the optimized SLN formula. The drug loadings, drug contents, incorporation efficiencies, and zeta potential for the SLN and NLC formulations are given in Table 3. The experimental entrapment efficiency of SLN and NLC was found to be 43.3% and 58.3%, respectively. As expected, the addition of liquid lipid increased CsA entrapment in the formulation. The particle size of SLN was 272.2 nm and that of NLC was 147.9 nm. Both formulations had a zeta potential of ∼20 mV.

#### 3.1.5 Microemulsion and nanoemulsion formulations

**Fig. 5:**
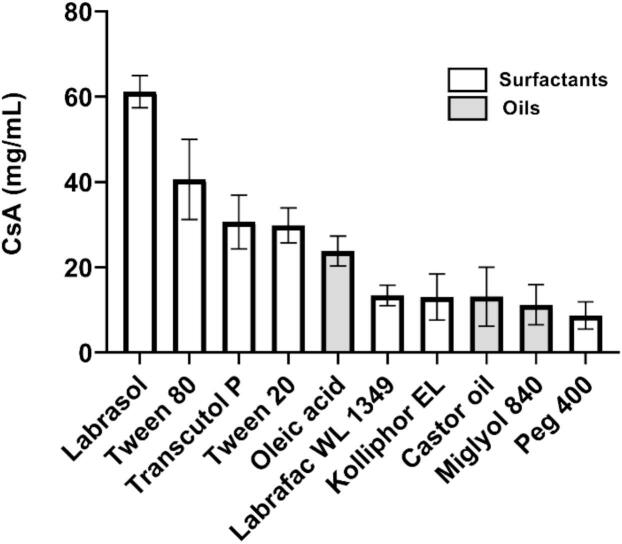
Solubility of CsA in different oils and surfactants.

The solubility profile of CsA in different surfactants, oils and solid lipids is presented in Fig. 5. Oleic acid and a Labrasol-Transcutol P mixture were selected respectively, as the oil and surfactant phases, to form the ME, given their ability to solubilize CsA.

**Fig. 6:**
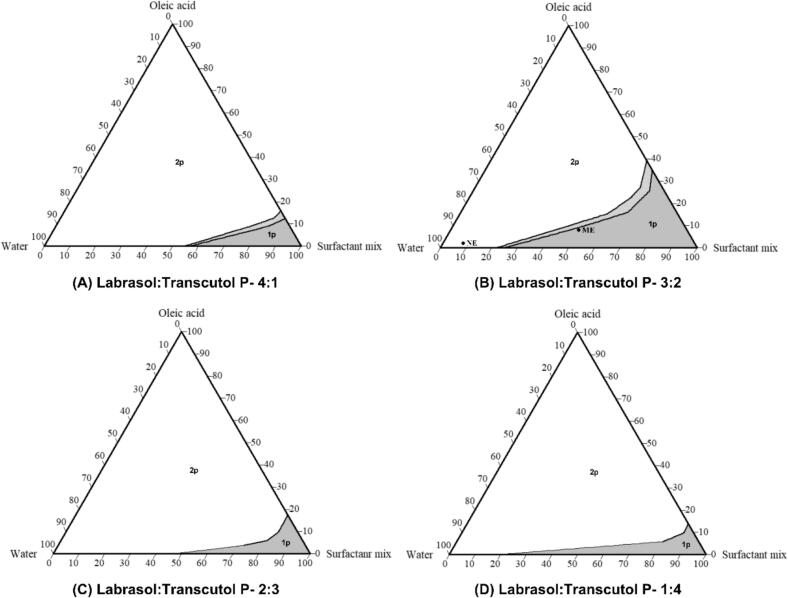
Ternary phase diagram for oleic acid–Labrasol:Transcutol P–water system; Various regions: dark-gray: one phase area, light-gray: meta stable area, white: two phase area.

Ternary phase diagrams for an oleic acid, Labrasol-Transcutol P – at 4:1, 3:2, 2:3, and 1:4 ratios – and water system are shown in Fig. 6. Labrasol-Transcutol P at 3:2 ratio was selected as the surfactant system because it produced stable ME along with sufficient solubility for the CsA in the corresponding ME compositions. The final ME composition was 8% *v*/v oleic acid, 50% v/v surfactant system and 42% v/v water (Fig. 6B). The ME was formed spontaneously yielding a transparent formulation. The ME was subjected to freeze-thaw cycles (−20 to 25 °C) of 24 h for 1 week to assess physical instability; however, the ME showed no phase separation or precipitation. The NE was selected from an emulsion region containing 2% v/v oleic acid, 8% v/v surfactant system and 90% v/v water (Fig. 6B). The NE was obtained by subjecting this emulsion to probe ultrasonication (Section 2.3.5) to reduce the globule size.

The drug loadings, drug contents, incorporation efficiencies, and zeta potential for the ME and NE formulations are given in Table 3. Both formulations showed >90% entrapment efficiency. The size of ME was 105.8 nm whereas NE were larger with a size of 202.8 nm.

#### 3.1.6 Morphology characterization

**Fig. 7:**
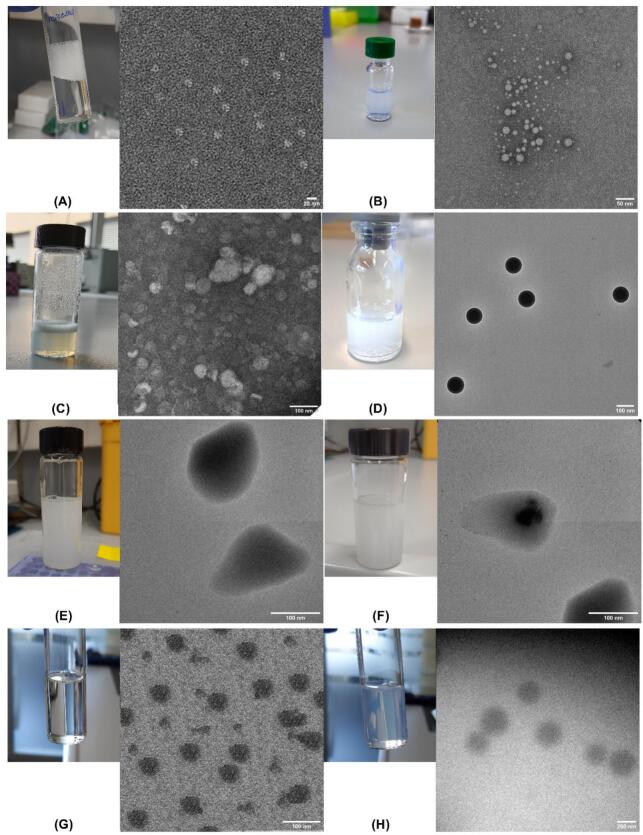
TEM image of CsA nanoformulations (2 mg/mL), (A) TPGS micelles, (B) mPEG-dihexPLA micelles, (C) liposomes, (D) Resomer® RG 503H (PLGA) nanoparticles, (E) solid lipid nanoparticles, (F) nanostructured lipid carriers, (G) microemulsion, (H) nanoemulsion.

CsA loaded nanoformulations were characterized to determine their size using DLS (Table 3). All CsA loaded micelle formulations presented uniform nanometer sizes. The TEM micrographs of the optimized nanoformulations are shown in Fig. 7. TEM morphology results were consistent with the DLS data.

#### 3.1.7 Nanoformulation stability

CsA content in the nanoformulations (stored at 4 °C) was quantified over a period of 4 weeks using UHPLC-MS/MS at different time points together with as assessment of any physicochemical changes (e.g. size, precipitation, assay, etc.). The results showed that all nanoformulations were stable for at least 4 weeks with no changes in physicochemical characteristics (**Supplementary Data, Section 8 – Fig. S3)**.

### 3.2 Evaluation of CsA delivery from the nanoformulations to porcine skin in vitro

#### 3.2.1 CsA cutaneous deposition and transdermal permeation

**Fig. 8:**
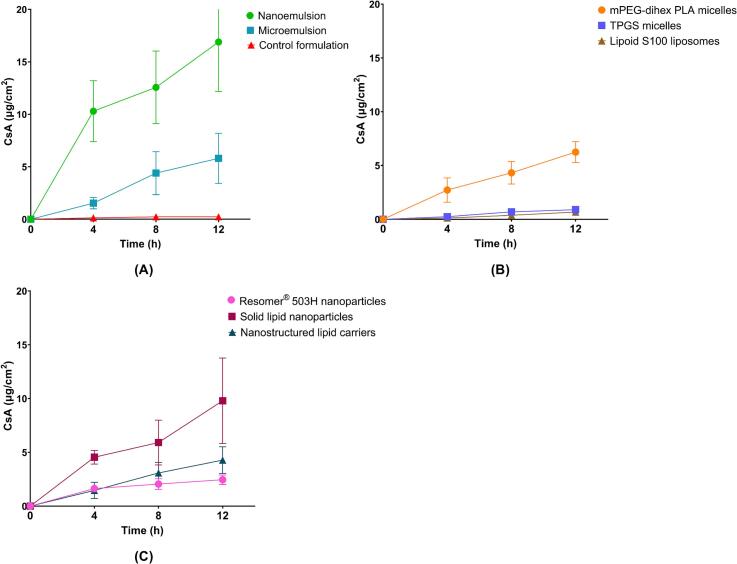
Cutaneous deposition of CsA from different nanoformulations and the control formulation (CsA in PG), (A) CsA deposition from microemulsion, and nanoemulsion and control formulations, (B) CsA deposition from mPEG-dihexPLA micelles, TPGS micelles, and Lipoid S100 liposomes, (C) CsA deposition from Resomer® 503H nanoparticles, solid lipid nanoparticles, and nanostructured lipid carriers. Mean ± SD (*n* = 6).

The concentration of CsA in the receiver compartment following application of each nanoformulation for 12 h was below the LOQ of the UHPLC-MS/MS method (< 4.9 ng/mL). CsA skin deposition was studied at 4, 8, and 12 h. As shown in Fig. 8, the NE showed the highest skin deposition at all time points followed by SLN and mPEG-dihexPLA micelles among all the nanoformulations and with respect to the control formulation (2 mg/mL CsA in propylene glycol). CsA delivery from mPEG-dihexPLA micelles was slightly lower than that observed in our previous study (6.3 ± 1.0 μg/cm^2^ and 8.7 ± 1.8 μg/cm^2^, respectively) but was consistent with the difference in CsA concentration (2 mg/mL and 5 mg/mL, respectively) (Lapteva et al., 2014).There was a significant difference in CsA skin deposition between NE and all other formulations (**Supplementary Data, Section 10.1 – Table S7**). TPGS micelles and Lipoid S100 liposomes performed poorly. Both nanoformulations only showed significantly higher deposition than the control formulation at 12 h (ANOVA; *p* < 0.001).

CsA delivery from the nanoformulations and the control after 12 h and the ratio of delivery from the nanoformulations to the control are shown in Table 5.

**Table 5:** Ranking of the cutaneous deposition of CsA achieved by nanoformulations after application for 12 h.

Formulation	CsA cutaneous deposition (μg/cm^2^)	Ratio of CsA deposition - Nanoformulation:Control
Nanoemulsion	16.89 ± 4.71	73.4
Solid lipid nanoparticles	9.80 ± 3.97	42.6
mPEG-dihexPLA micelles	6.25 ± 0.98	27.2
Microemulsion	5.81 ± 2.39	25.3
Nanostructured lipid carriers	4.28 ± 1.24	18.6
Resomer® 503H nanoparticles	2.46 ± 0.44	10.7
TPGS micelles	0.90 ± 0.21	3.9
Lipoid S100 liposomes	0.67 ± 0.16	2.9
Control formulation	0.23 ± 0.06	1

#### 3.2.2 CsA cutaneous biodistribution

**Fig. 9:**
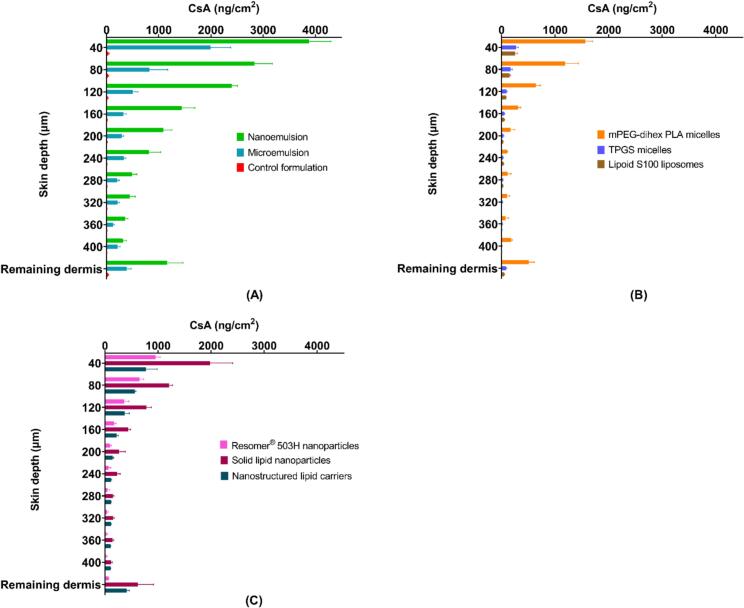
Cutaneous biodistribution of CsA from different nanoformulations. (A) CsA biodistribution profile from microemulsion, nanoemulsion, and control formulation, (B) CsA biodistribution profile from mPEG-dihexPLA micelles, TPGS micelles, and Lipoid S100 liposomes, (C) CsA biodistribution profile from Resomer® 503H nanoparticles, solid lipid nanoparticles, and nanostructured lipid carriers. Mean ± SD (*n* = 6).

Biodistribution studies enabled determination of the amounts of CsA present as a function of depth in the different skin regions and confirmed that greater amounts of CsA were predominantly present in the epidermal region. Fig. 9A compares the emulsion-based nanosystems and the control formulation. Greater amounts of CsA were observed at each depth following application of the NE as compared to the ME and the control formulation, despite the fact that the ME had a much higher surfactant content (50% vs 8%) and a smaller particle size. The ME was outperformed most probably due to the higher thermodynamic activity of CsA in the NE. Furthermore, the NE was also able to deliver CsA to the deeper layers of the skin. For example, in the 80–120 μm skin section, CsA deposition for NE, ME and control formulations was 2401.50 ± 104.17 ng/cm^2^, 502.31 ± 100.81 ng/cm^2^, and 21.77 ± 11.11 ng/cm^2^, respectively. There was a significant difference in CsA delivery between NE and ME formulations at all skin depths (**Supplementary Data, Section 10.2 – Table S8**).

Vesicle-based nanosystems are compared in Fig. 9B: mPEG-dihexPLA micelles delivered greater amounts of CsA in epidermal and dermal regions than TPGS micelles and Lipoid S100 liposomes. The mPEG-dihexPLA being more lipophilic in nature and having lower CMC (1.6 μM) (Lapteva et al., 2014) than TPGS (20 μM) (Sadoqi et al., 2009) might have resulted in more CsA loading per micelle thereby increasing the concentration gradient for CsA partitioning into the skin after micelle disassembly. The significant difference in CsA delivery between mPEG-dihexPLA micelles and TPGS micelles or Lipoid S100 liposomes was observed at all skin depths (*P* < 0.05, ANOVA). However, there was no significant difference between the biodistribution profiles of TPGS micelles and liposomes.

Cutaneous deposition of CsA following application of polymeric and lipid-based nanoparticles is compared in Fig. 9C. SLN delivered greater amounts of CsA to the epidermal and dermal regions compared to polymeric nanoparticles and NLC. There was no significant difference between PLGA nanoparticles and NLC. Despite having a smaller particle size and a higher lipid content which increased the CsA loading in NLC, CsA skin deposition from NLC was lower than that from SLN. Solid lipid crystallinity affects the release properties of SLN (Pawar et al., 2012; Chantaburanan et al., 2017). The transition from a high-energy state to low energy crystalline state aids drug release. Solid lipids in NLC are stabilized by liquid lipid, slowing down this transition and hence the drug release (Jaiswal et al., 2016). Water evaporation upon formulation application to the skin leads to lipid modifications in the SLN resulting in drug expulsion and increased penetration into the skin (Borges et al., 2020; Jenning et al., 2000; Surber and Knie, 2018). In the case of polymeric NP, the molecular weight of PLGA plays an important role in determining the drug release rate which increases with a decrease in the molecular weight of the polymer (Kumskova et al., 2020). Resomer® RG 502H (PLGA; MW: 7–17 kDa) might have shown more CsA release; however, the entrapment efficiency was too low and hence Resomer® RG 503H (PLGA; MW: 24–38 kDa) was selected. There was a significant difference in CsA delivery between SLN and both NLC and polymeric nanoparticle formulations at all skin depths (*p* < 0.05, ANOVA) whereas, there was no significant difference between the polymeric nanoparticles and NLC formulations.

**Fig. 10:**
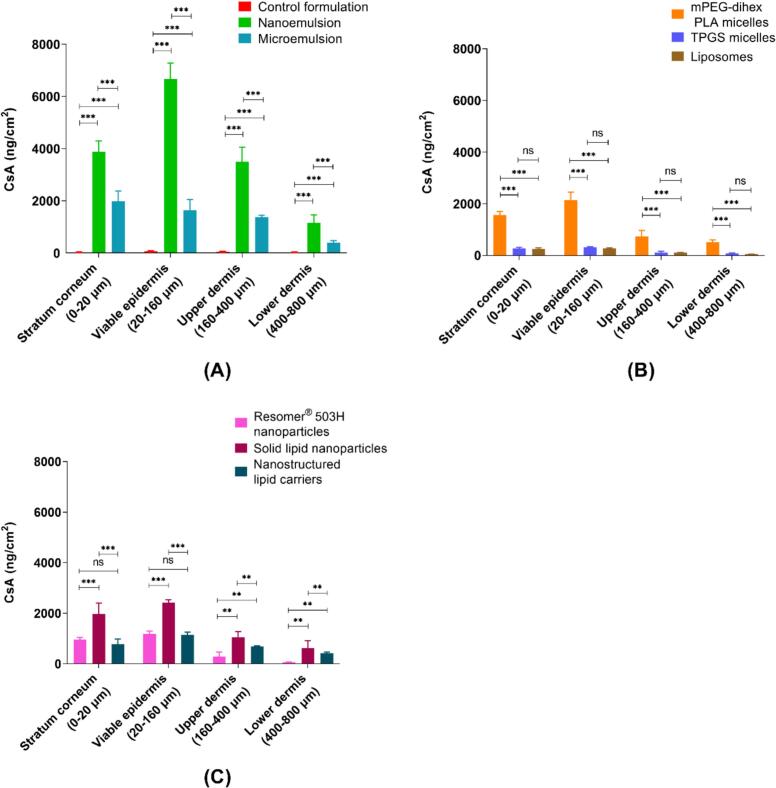
Cutaneous biodistribution of CsA as a function of the anatomical regions of the skin (stratum corneum + viable epidermis, lower and upper dermis) after formulation application for 12 h; (A) CsA distribution in different skin layers from the microemulsion, nanoemulsion, and control formulation, (B) CsA distribution in different skin layers from mPEG-dihexPLA micelles, TPGS micelles, and Lipoid S100 liposomes, (C) CsA distribution in different skin layers from Resomer® 503H nanoparticles, solid lipid nanoparticles, and nanostructured lipid carriers. *P*-values were calculated using ANOVA; statistically differences were denoted by asterisks (ns – no significant difference, **P < 0.05 and *** *P* < 0.001).

These results are summarized in Fig. 10 as a function of the amounts of CsA present in the different anatomical regions of the skin (stratum corneum + viable epidermis, upper and lower dermis). All nanoformulations preferentially delivered CsA to the epidermis (stratum corneum and viable epidermis). NE delivered the highest amounts of CsA to all skin layers. The amount of CsA delivered by NE to upper dermis was comparable or even superior to the amount delivered to the viable epidermis by many of the other nanoformulations. The control formulation performed very poorly. Detailed statistical analysis data for all nanoformulations is provided in the **Supplementary data (Section 10.2 – Table S9**). Among vesicular nanosystems, mPEG-dihexPLA micelles performed quite well, delivering 2139.40 ± 309.82 ng/cm^2^ of CsA to viable epidermis, which was comparable to that observed with SLN (2425.48 ± 112.07 ng/cm^2^). With relatively lower dermal delivery, these nanosystems along with other particulate nanosystems can be quite useful in treating epidermal skin conditions as higher drug delivery in the target area (epidermis) would be ensured with minimum exposure to the dermal region and hence minimum systemic absorption. The other vesicular nanosystems, TPGS micelles and Lipoid S100 liposomes, although delivering high amounts of CsA to the viable epidermis as compared to the control formulation, performed poorly in delivering CsA as compared to other nanosystems.

#### 3.2.3 CsA follicular delivery in porcine skin

**Fig. 11:**
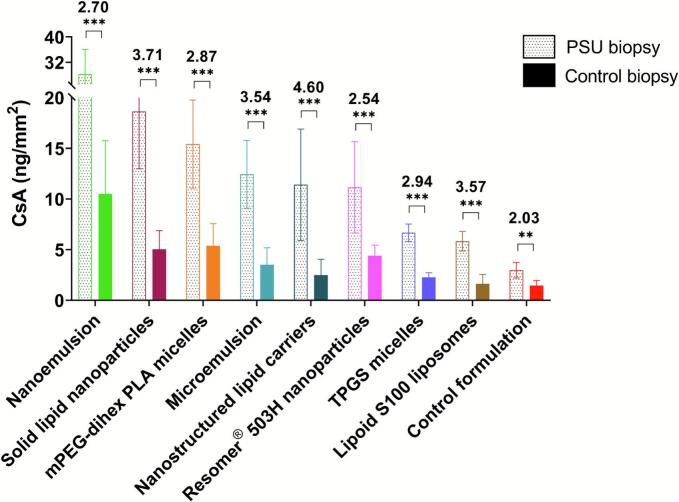
Comparison of CsA delivery from different nanoformulations to the PSU-containing and PSU-free skin biopsies. (***– *P* < 0.001; **– P < 0.05; Follicle targeting ability (FTA) value is expressed above the SD bar. Mean ± SD (*n* = 6).

There is ongoing interest in the development of formulations that enable preferential drug delivery to the pilosebaceous unit (PSU - consisting of hair follicle, hair shaft, arrector pili muscle, and sebaceous gland). Although PSU only constitute 0.1% of the total skin surface area, they can be sites for different diseases (e.g. mycoses, acne vulgaris, folliculitis, seborrheic dermatitis) making preferential targeting of the PSU by nanocarriers of significant clinical interest (Lapteva et al., 2015; Fresta et al., 2020; Chourasia and Jain, 2009). Hence, it was decided to compare the ability of the different nanoformulations to deliver CsA to PSU-containing and PSU-free biopsies (i.e. control) – recognizing that follicular delivery of CsA is not a key objective for its own therapeutic indications (Fig. 11). For each of the tested nanoformulations, CsA delivery to the PSU-containing biopsy was significantly higher (*P* < 0.05, ANOVA) than that to the control biopsy. Upon topical application, the nanoformulation can accumulate in the volume between the hair shaft and the PSU epidermal layer and thereby increase the amount of drug present in the PSU-containing biopsy; this would act as a drug depot in vivo. The follicle targeting ability (FTA) was defined as the ratio of the amount of CsA in the PSU-containing and PSU-free biopsies.

#### 3.2.4 Understanding CsA delivery from the nanoformulations

The cutaneous delivery studies showed how different nanoformulations behaved when in contact with skin and their ability to deliver CsA. There are numerous variables that might potentially affect the cutaneous delivery of a molecule from a specific nanocarrier. These include the size of the molecule, its physicochemical properties, nanocarrier attributes, excipients used and their physicochemical properties, method of preparation, and of course the condition of the skin (i.e. healthy or diseased), to name but a few. In this study, it was attempted to standardize conditions so to facilitate comparison of the different nanocarriers and to identify the standout attributes of the nanocarriers that resulted in better cutaneous delivery of CsA. The results clearly point to the pivotal role of thermodynamic activity and the relative affinity of CsA for the formulation and the skin. The NE outperformed the ME despite having a lower oil and surfactant content – the latter might also have been considered to afford some form of penetration enhancement. Similarly, despite having a lower lipid content and lower entrapment efficiency, SLN outperformed the NLC. Excess surfactant or lipid/oil in the nanoformulation can increase the solubility of the drug in the nanoformulation itself and reduce its thermodynamic activity which in turn can reduce drug partitioning into the skin. Conversely, high thermodynamic activity of the drug in the formulation will increase the partitioning of the drug into the skin thereby increasing its concentration at the stratum corneum/formulation interface and hence the concentration gradient and flux – resulting in improved cutaneous bioavailability and distribution to the deeper skin layers.

The type of excipient used is another influencing factor. Although both micellar preparations had the smallest sizes compared to other nanosystems, mPEG-dihexPLA micelles outperformed TPGS micelles with respect to the cutaneous delivery of CsA. To achieve 0.2% *w*/*v* CsA content in formulation, 0.82 mM mPEG-dihexPLA was required compared to 6.61 mM TPGS for the same loading. This might have resulted in increased CsA concentration in mPEG-dihexPLA micelles resulting in enhanced CsA delivery. Having a higher molecular weight, mPEG-dihexPLA (MW, 6080 g/mol) micelles could not cross the stratum corneum. Micelles deform/disaggregate at the skin surface, unloading the drug cargo, potentially resulting in transient local supersaturation, and thereby facilitating partitioning into the skin especially from the inter-corneocyte and inter-cluster regions (Lapteva et al., 2014). Similar mechanisms could be involved in the case of liposomal formulations with vesicles again rupturing at the surface to create a high local drug concentration resulting in increased drug partitioning into the skin. In the case of polymeric NP, drug diffusion and polymer erosion are the two main mechanisms of drug release from PLGA based systems (Han et al., 2016). PLGA is a bulk eroding polymer degraded through the hydrolysis of its polyester backbone. This hydrolysis is catalyzed in the presence of an acid (Hines and Kaplan, 2013). Drug diffusion along with drug release from polymer erosion in acidic pH at the skin surface (pH ∼5) (Lambers et al., 2006) explains CsA delivery from these systems into the skin. Interestingly, the measured size of the nanocarriers in the formulations was certainly not pivotal as demonstrated by results seen with the TPGS micelles, which had the smallest size but did not display the best delivery, which was seen with NE, which were ∼ 20 times larger.

Skin structures also play a crucial role in cutaneous drug delivery. All nanoformulations along with the control formulation delivered CsA preferentially to the PSU-containing biopsies as compared to equivalent PSU-free regions. Upon application to the skin, the nanoformulations can accumulate in hair follicles and create a drug depot (Pelikh and Keck, 2020). The hydrophobic environment in the hair follicle due to sebum further aids in this mechanism (Jain et al., 2016). To assess how CsA is released and distributed from follicular pathway or transcellular pathway, imaging studies need to be performed to establish evolution of drug biodistribution in the skin as a function of time post-delivery. This remains the future perspective of this study, that can be performed using novel techniques such as MS-DESI imaging (Quartier et al., 2021b).

## 4 Conclusion

In this comprehensive study, a series of different nanoformulations were developed using a DoE approach, optimized and characterized. Subsequent delivery experiments indicated that although all the nanoformulations significantly increased cutaneous bioavailability of CsA as compared to the control and without any transdermal permeation – thus, resulting in targeted delivery to the skin and reducing the risk of systemic side effects of the drug in vivo – there were significant differences in their behaviour. Among the tested formulations, NE showed the highest cutaneous CsA delivery followed by SLN and mPEG-dihexPLA micelles. Time-based skin deposition studies revealed that CsA delivery was directly proportional to the application time. In addition to comparing the ability of the different nanoformulations to deliver CsA to the skin, delivery to the PSU was also measured and demonstrated the capacities of the nanoformulations to preferentially deliver CsA to these structures.

Head-to-head comparison of the different nanosystems under controlled conditions enabled us to understand the importance of thermodynamic activity of formulations in delivering CsA to the skin. Formulations with relatively high thermodynamic activity such as NE showed better payload delivery compared to those with lower activity such as ME, for a given exposure time. The thermodynamic activity should obviously be balanced in a way to have better delivery efficiency combined with appropriate physical stability during the shelf life. Furthermore, the measured size of the nanocarriers was not pivotal to CsA delivery since the TPGS micelles, which had the smallest measured size, did not have the highest CsA delivery. However, it is clear that the size of nanocarriers that retain their structural integrity upon contact with the skin will determine their access to appendageal structures.

Future fundamental studies will investigate other challenging APIs, with different molecular properties, and test them with similar panels of nanocarriers to determine whether the same trends are observed or whether specific drug characteristics might affect nanocarrier performance with respect to cutaneous and follicular delivery.

Ciclosporin A (CsA) has many therapeutic indications in dermatology but there are no approved topical formulations for its application directly to the skin (Pandey et al., 2020; Arora et al., 2017). From a clinical perspective, these CsA results should obviously be confirmed in vivo with diseased skin as histopathological changes might alter delivery from the nanocarriers, but they provide a good starting point for the development of a clinically relevant topical formulation.

## CRediT authorship contribution statement

**Aditya R. Darade:** Writing – review & editing, Writing – original draft, Validation, Methodology, Investigation, Data curation. **Maria Lapteva:** Writing – review & editing, Supervision, Methodology, Investigation. **Yogeshvar N. Kalia:** Writing – review & editing, Supervision, Resources, Funding acquisition, Conceptualization.

## Declaration of competing interest

The authors declare that they have no known competing financial interests or personal relationships that could have appeared to influence the work reported in this paper.

## Data Availability

Data will be made available on request.

## Appendix A Supplementary data

Supplementary material Image 1
