# Supplementary material for: Comparing the efficacy of nanocarriers for cutaneous and follicular delivery of poorly water-soluble molecules: A case study with ciclosporin A
Source: Int J Pharm X. 2026 Feb 10;11:100505. doi: 10.1016/j.ijpx.2026.100505 (PMC12914692; doi:10.1016/j.ijpx.2026.100505)
Supplement: Supplementary file 1 — Supplementary material [file mmc1.docx]

**SUPPLEMENTARY DATA**

**Comparing the efficacy of nanocarriers for cutaneous and follicular delivery of poorly water-soluble molecules: A case study with ciclosporin A**

**Aditya R. Darade^a,b^, Maria Lapteva^a,b^, Yogeshvar N. Kalia^a,b*^**

^a^ School of Pharmaceutical Sciences, University of Geneva, CMU, 1 rue Michel-Servet, 1211, Geneva 4, Switzerland

^b^ Institute of Pharmaceutical Sciences Western Switzerland, University of Geneva, Geneva, Switzerland

**Corresponding author**

Prof. Yogeshvar N. Kalia

School of Pharmaceutical Sciences, University of Geneva

CMU, 1 rue Michel-Servet, 1211, Geneva 4, Switzerland

Tel (dir): +41 (0)22 379 3355

Email: [yogi.kalia@unige.ch](mailto:yogi.kalia@unige.ch)

**1. Validation of UPLC-MS/MS analytical method**

1.1 Specificity

**Figure S1** presents the chromatograms obtained for the CsA standard. The method was considered to be specific for CsA (eluted at 3.2 min). No response was observed from endogenous skin compounds present in the permeation samples and skin extracts.


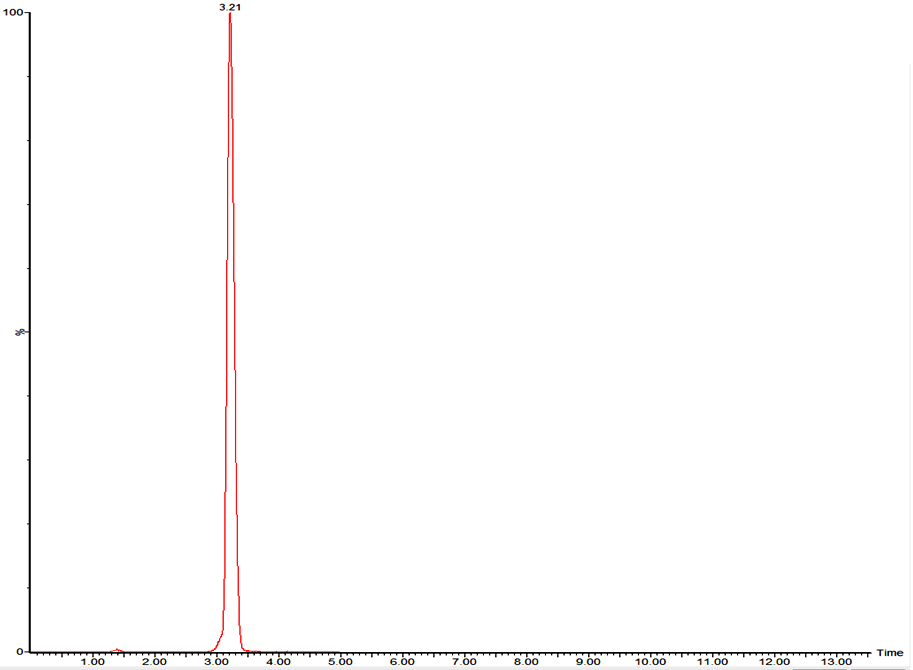


**Figure S1.** CsA chromatogram (100 ng/mL)

1.2 Linearity

The method was linear in the concentration range of 5 to 500 ng/mL with an R^2^ of 0.9999

1.3 Limit of detection and limit of quantification

The limit of detection (LOD) and limit of quantification (LOQ) were determined using the linear regression method and found to be 1.61 ng/mL and 4.90 ng/mL, respectively.

1.4 Precision and accuracy

Intra- and inter-day precision and accuracy were determined using 5, 50, and 100 ng/mL of CsA solutions in skin extracts. **Table S1** shows a summary of intra- and inter-day accuracy and precision for the method which complied with ICH guidelines (Q2(R1)).

**Table S1.** Intra and Inter-day accuracy and precision for the CsA UPLC-MS/MS method

| **CsA ng/mL** | **Intra-day** | | | **Inter-day 1** | | | **Inter-day 2** | | |
| --- | --- | --- | --- | --- | --- | --- | --- | --- | --- |
|  | **CsA (mean ± SD) in ng/mL** | **RSD**  **(%)** | **Recovery**  **(%)** | **CsA (mean ± SD) in ng/mL** | **RSD**  **(%)** | **Recovery**  **(%)** | **CsA (mean ± SD) in ng/mL** | **RSD**  **(%)** | **Recovery**  **(%)** |
| 5 | 4.83 ± 0.31 | 6.41 | 96.6 | 4.89 ± 0.23 | 4.70 | 97.8 | 4.92 ± 0.29 | 5.89 | 98.4 |
| 50 | 50.01 ± 1.31 | 2.62 | 100.02 | 49.85 ± 0.97 | 1.95 | 99.70 | 49.82 ± 1.12 | 2.25 | 99.64 |
| 100 | 99.91 ± 1.03 | 1.03 | 99.91 | 99.28 ± 1.55 | 1.56 | 99.28 | 98.96 ± 1.93 | 1.95 | 98.96 |

**2. Filter adsorption studies**

Various 0.22 µm syringe filters such as nylon, PVDF, PTFE, and CA were screened for drug adsorption. Known concentrations (low, middle, and high) of CsA solutions were passed through filters (n=3) and analyzed for drug adsorption. PTFE filters showed negligible adsorption and hence was considered suitable for processing CsA samples (**Table S2**).

**Table S2.** CsA filter adsorption at different concentrations

| **Conc.**  **(ng/mL)** | **Recovery**  **(% ± SD )** | | | |
| --- | --- | --- | --- | --- |
|  | **Nylon** | **Cellulose acetate** | **PVDF** | **PTFE** |
| 500 | 45.89 ± 3.27 | 90.28 ± 0.72 | 21.74 ± 3.33 | 99.32 ± 0.25 |
| 100 | 52.04 ± 1.57 | 92.10 ± 1.70 | 25.85 ± 4.35 | 100.20 ± 1.35 |
| 50 | 50.71 ± 2.02 | 92.37 ± 1.07 | 17.05 ± 2.44 | 99.47 ± 0.38 |

**3. Validation of CsA extraction procedure from skin samples**

50 µL of known concentrations (2, 4 and 20 µg/mL) of CsA in acetone were applied to porcine skin samples (n=3; area=0.8 cm^2^). Acetone was allowed to evaporate and the skin samples were cut into small pieces and extracted with 2 mL methanol for 4 h to yield theoretical concentrations of 5, 50, 200 and 500 ng/mL of CsA. Extracts were then analyzed by UPLC-MS/MS method.

The extraction process was considered acceptable given the reproducible recovery. Results for extraction procedure validation are shown in **Table S3**.

**Table S3.** Validation of CsA extraction efficacy from skin samples

| **[CsA] theoretical**  **(ng/mL)** | **[CsA] recovered**  **(ng/mL±SD)** | **% Recovery** |
| --- | --- | --- |
| 50 | 49.47 **±** 3.19 | 98.94 |
| 100 | 99.25 **±** 2.21 | 99.25 |
| 500 | 487.35 **±** 1.95 | 97.47 |

**4. Validation of wash process**

Different nanoformulations were applied to porcine skin (n = 3) in concentrations similar to the delivery experiments and immediately removed by washing under running water and wiping with cotton buds and dried with soft tissue paper. The skin samples were cut into small pieces, extracted, and analyzed by UPLC-MS/MS. Concentrations of CsA were below the LOD in the extraction samples suggesting the efficacy of the wash procedure to remove excess surface formulation.

**Table S4.** Validation of CsA wash process from porcine skin samples (n=3; experimental values are presented as the Mean **±** SD)

| **Formulation** | **[CsA]_experimental_**  **(ng/mL)** |
| --- | --- |
| TPGS micelles | 0.49 ± 0.22 |
| mPEG-dihexPLA micelles | 0.19 ± 0.16 |
| Lipoid S100 liposomes | 0.38 ± 0.24 |
| Resomer® 503H nanoparticles | 0.32 ± 0.26 |
| Solid lipid nanoparticles | 0.83 ± 0.39 |
| Nanostructured lipid carriers | 0.64 ± 0.34 |
| Microemulsion | 0.26 ± 0.18 |
| Nanoemulsion | 0.92 ± 0.29 |

**5. Determination of entrapment efficacy of CsA nanoformulations**

The entrapment efficiency was determined by using Amicon Ultra-0.5 mL centrifugal filters with pore size cut off 3 kDa (nominal pore size 0.3 nm). Samples were centrifuged to remove excess or precipitated drug. The supernatant was then loaded in Amicon filters and centrifuged at 5,000 rpm for 10 min. The pore size of 3 kDa is small enough to prevent the passage of nanosystems, but allowing free drug (solubilized form) to pass. This method was validated by using standard CsA solutions. Various known concentrations were passed through centrifugal filters and recovery was analyzed by UPLC-MS/MS. Concentrations below 100 ng/mL, showed a dramatic reduction in recovery due to possible adsorption on membrane plastic or membrane itself. Higher concentrations showed acceptable and reproducible recovery.

**Table S5.** Validation of CsA ultrafiltration from Amicon Ultra-0.5 mL centrifugal filters (experimental values are presented as the Mean **±** SD)

| **[CsA]_Initial_**  **(ng/mL)** | **[CsA]_Post-filtration_**  **(ng/mL)** | **% Recovery** |
| --- | --- | --- |
| 10 | 0.91 **±** 0.04 (<LOD) | 9.1 |
| 50 | 19.59 **±** 6.81 | 39.18 |
| 100 | 63.02 **±** 4.18 | 63.02 |
| 500 | 469.94 **±** 3.77 | 93.99 |
| 1000 | 971.64 **±** 5.14 | 97.16 |

**6. Experimental DoE**

**Table S6.** Experimental DoE for the nanoformulations

| **TPGS micelles** | | | | | | | |  | **Lipoid S100 liposomes** | | | | | | | | |
| --- | --- | --- | --- | --- | --- | --- | --- | --- | --- | --- | --- | --- | --- | --- | --- | --- | --- |
| Run | Factor A: CsA mg/mL | | | Factor B: TPGS mg/mL | | Response % Entrapment efficiency | |  | Run | | | Factor A: CsA mg/mL | | Factor B: Lipoid S100 mg/mL | | | Response % Entrapment efficiency |
| 1 | 4.17 | | | 10 | | 71.80 | |  | 1 | | | 2.50 | | 45.00 | | | 100 |
| 2 | 4.17 | | | 10 | | 76.10 | |  | 2 | | | 2.50 | | 45.00 | | | 100 |
| 3 | 8.24 | | | 10 | | 31.50 | |  | 3 | | | 1.00 | | 10.00 | | | 78.12 |
| 4 | 7.05 | | | 15 | | 43.37 | |  | 4 | | | 2.50 | | 45.00 | | | 100 |
| 5 | 4.17 | | | 2.93 | | 20.89 | |  | 5 | | | 2.50 | | 45.00 | | | 100 |
| 6 | 7.05 | | | 5 | | 22.30 | |  | 6 | | | 4.00 | | 80.00 | | | 100 |
| 7 | 1.29 | | | 15 | | 100 | |  | 7 | | | 2.50 | | 10.00 | | | 31.82 |
| 8 | 4.17 | | | 17.07 | | 100 | |  | 8 | | | 4.62 | | 45.00 | | | 50.64 |
| 9 | 3.00 | | | 10 | | 100 | |  | 9 | | | 1.00 | | 80.00 | | | 100 |
| 10 | 0.10 | | | 10 | | 100 | |  | 10 | | | 4.00 | | 10.00 | | | 10.29 |
| 11 | 4.17 | | | 10 | | 75.90 | |  | 11 | | | 2.50 | | 94.50 | | | 100 |
| 12 | 4.17 | | | 10 | | 73.40 | |  | 12 | | | 0.38 | | 45.00 | | | 100 |
| 13 | 1.29 | | | 5 | | 100 | |  | 13 | | | 2.50 | | 45.00 | | | 100 |
|  | | | | | | | | | | | | | | | | | |
| **Resomer® RG 503H (PLGA) nanoparticles** | | | | | | | | | | | | | | | | | |
| Run | | Factor A: CsA mg/mL | | | Factor B: PLGA mg/mL | | | | | Factor C: % Tween 20 | | | Factor D: % PVP | | | Response % Entrapment efficiency | |
| 1 | | 3.00 | | | 70.00 | | | | | 2.00 | | | 5.00 | | | 84.72 | |
| 2 | | 1.00 | | | 70.00 | | | | | 2.00 | | | 5.00 | | | 89.10 | |
| 3 | | 1.00 | | | 70.00 | | | | | 0.00 | | | 5.00 | | | 91.32 | |
| 4 | | 3.00 | | | 70.00 | | | | | 0.00 | | | 5.00 | | | 76.44 | |
| 5 | | 3.00 | | | 20.00 | | | | | 0.00 | | | 1.00 | | | 25.81 | |
| 6 | | 2.00 | | | 45.00 | | | | | 3.00 | | | 3.00 | | | 69.34 | |
| 7 | | 3.00 | | | 70.00 | | | | | 2.00 | | | 1.00 | | | 78.91 | |
| 8 | | 4.00 | | | 45.00 | | | | | 1.00 | | | 3.00 | | | 35.09 | |
| 9 | | 3.00 | | | 20.00 | | | | | 2.00 | | | 1.00 | | | 21.68 | |
| 10 | | 3.00 | | | 20.00 | | | | | 0.00 | | | 5.00 | | | 23.19 | |
| 11 | | 1.00 | | | 20.00 | | | | | 2.00 | | | 5.00 | | | 65.84 | |
| 12 | | 1.00 | | | 20.00 | | | | | 0.00 | | | 5.00 | | | 41.44 | |
| 13 | | 2.00 | | | 95.00 | | | | | 1.00 | | | 3.00 | | | 91.35 | |
| 14 | | 1.00 | | | 20.00 | | | | | 0.00 | | | 1.00 | | | 46.82 | |
| 15 | | 3.00 | | | 70.00 | | | | | 0.00 | | | 1.00 | | | 74.04 | |
| 16 | | 1.00 | | | 70.00 | | | | | 2.00 | | | 1.00 | | | 92.52 | |
| 17 | | 3.00 | | | 20.00 | | | | | 2.00 | | | 5.00 | | | 21.73 | |
| 18 | | 1.00 | | | 70.00 | | | | | 0.00 | | | 1.00 | | | 93.11 | |
| 19 | | 1.00 | | | 20.00 | | | | | 2.00 | | | 1.00 | | | 61.93 | |
|  | | | | | | | | | | | | | | | | | |
| **Solid lipid nanoparticles** | | | | | | | | | | | | | | | | | |
| Run | | | Factor A: GMS mg/mL | | | | Factor B: Lipoid S100 mg/mL | | | | Factor C: % Tween 20 | | | | Response % Entrapment efficiency | | |
| 1 | | | 15.00 | | | | 0.50 | | | | 0.10 | | | | 49.81 | | |
| 2 | | | 48.52 | | | | 0.30 | | | | 1.55 | | | | 83.57 | | |
| 3 | | | 40.00 | | | | 0.10 | | | | 0.10 | | | | 80.22 | | |
| 4 | | | 27.50 | | | | 0.30 | | | | 1.55 | | | | 71.67 | | |
| 5 | | | 40.00 | | | | 0.50 | | | | 3.00 | | | | 81.08 | | |
| 6 | | | 27.50 | | | | 0.30 | | | | 1.55 | | | | 71.15 | | |
| 7 | | | 40.00 | | | | 0.50 | | | | 0.10 | | | | 83.49 | | |
| 8 | | | 40.00 | | | | 0.10 | | | | 3.00 | | | | 82.71 | | |
| 9 | | | 15.00 | | | | 0.10 | | | | 3.00 | | | | 47.33 | | |
| 10 | | | 27.50 | | | | 0.64 | | | | 1.55 | | | | 76.49 | | |
| 11 | | | 27.50 | | | | 0.30 | | | | 3.99 | | | | 78.56 | | |
| 12 | | | 15.00 | | | | 0.10 | | | | 0.10 | | | | 43.12 | | |
| 13 | | | 15.00 | | | | 0.50 | | | | 3.00 | | | | 49.01 | | |
| 14 | | | 6.48 | | | | 0.30 | | | | 1.55 | | | | 9.14 | | |

**7.** **Normal plot of residues for different nanosystems using DoE**

The normal plot of residuals is presented in **Figure S2**. It indicates whether the residuals follow a normal distribution or not. In case of normal distribution, the points will follow a straight line. Some moderate scatter can be expected even with normal distribution.


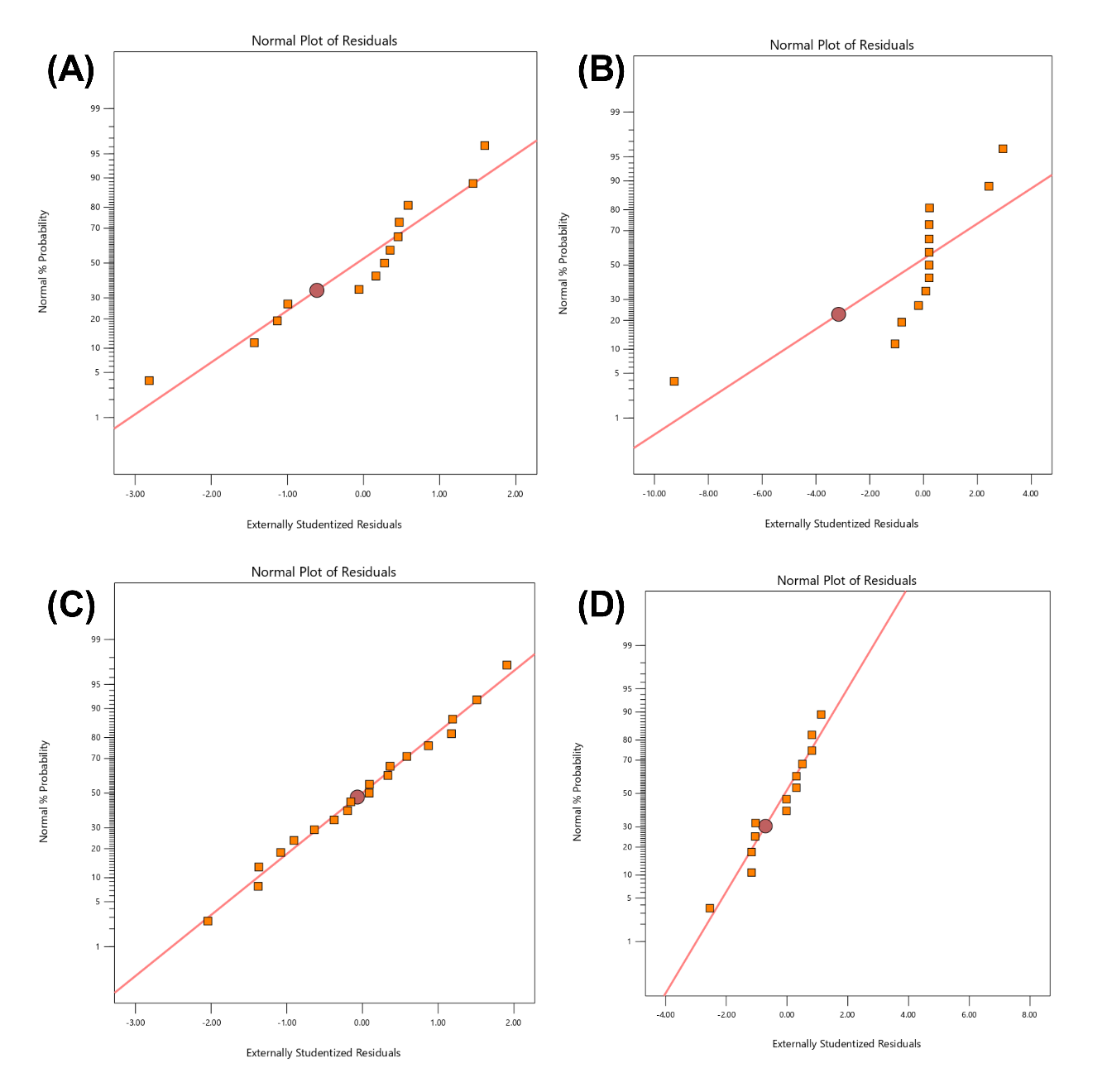


**Figure S2.** Normal plot of residues for DoE models of nanosystems, (A) micelles, (B) liposomes, (C) Resomer® 503H nanoparticles, (D) solid lipid nanoparticles

**8. Stability data (4 weeks) for nanoformulation**

CsA was assayed from nanoformulations at the day of preparation followed by week 2 and 4. Nanoformulations were also checked for particle size at random points in 4 weeks. No dramatic increase in size was observed (data not shown).


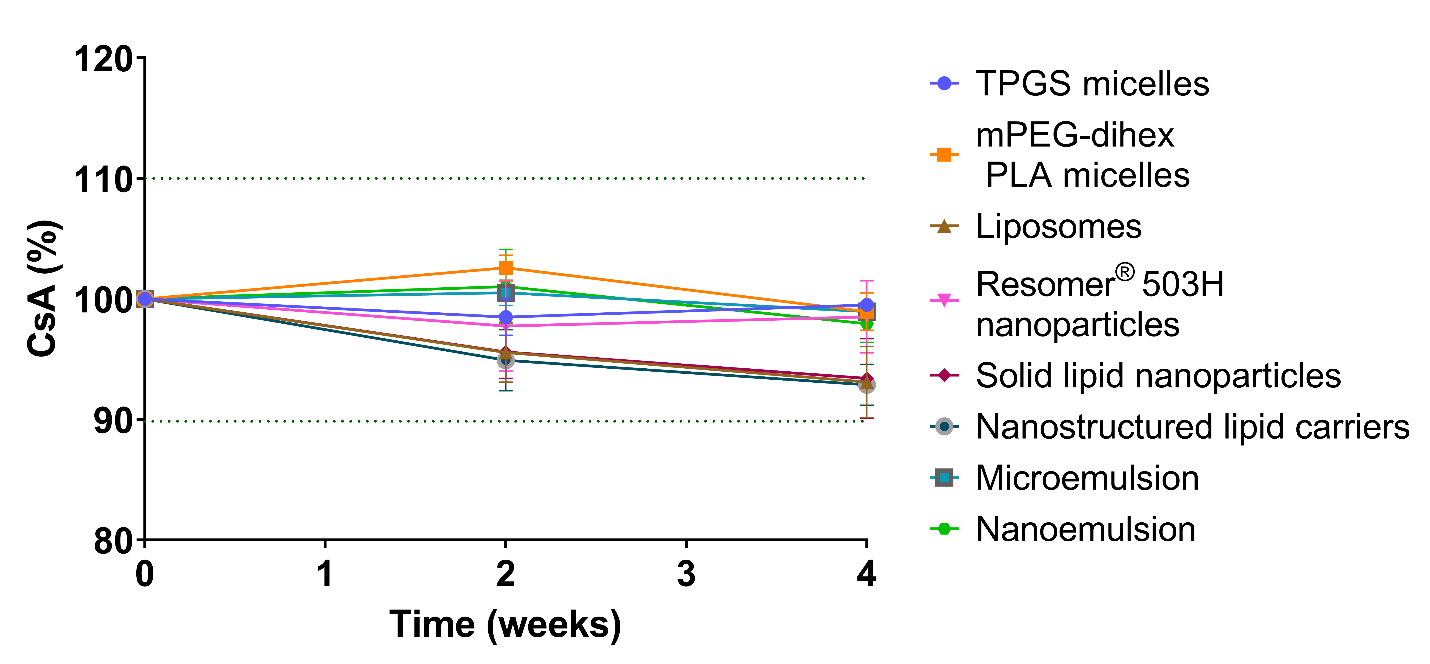


**Figure S3.** Stability (drug assay) plot of nanoformulations for 4 weeks

**9. PSU biopsy and control (PSU-free) biopsy obtained by 1 mm punch**


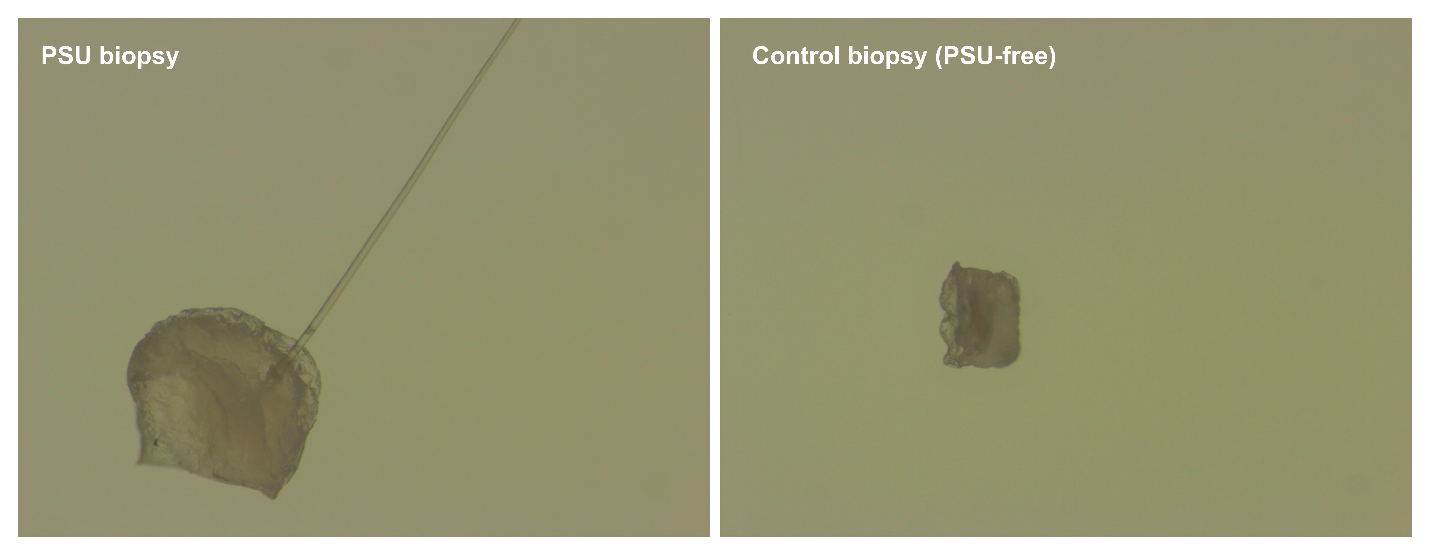


**Figure S4.** PSU biopsy and control biopsy (PSU-free)

**10. Statistical analysis**

Results were evaluated statistically using analysis of variance (ANOVA) one-way followed by the Tukey test for multiple comparisons or Student’s t test. The level of significance was fixed at α = 0.05.

*10.1 CsA deposition in porcine skin*

**Table S7.** P values for CsA delivery from different nanoformulations in skin deposition studies (12 h)

|  | **Control formulation** | **NE** | **ME** | **TPGS micelles** | **mPEG-dihexPLA micelles** | **Liposomes** | **PLGA NP** | **SLN** | **NLC** |
| --- | --- | --- | --- | --- | --- | --- | --- | --- | --- |
| **Control formulation** |  | <0.001 | <0.001 | <0.001 | <0.001 | <0.001 | <0.001 | <0.001 | <0.001 |
| **NE** |  |  | <0.001 | <0.001 | <0.001 | <0.001 | <0.001 | 0.018 | <0.001 |
| **ME** |  |  |  | <0.001 | 0.787 | <0.001 | 0.007 | 0.061 | 0.195 |
| **TPGS micelles** |  |  |  |  | <0.001 | 0.066 | <0.001 | <0.001 | <0.001 |
| **mPEG-dihexPLA micelles** |  |  |  |  |  | <0.001 | 0.007 | 0.098 | 0.142 |
| **Liposomes** |  |  |  |  |  |  | <0.001 | <0.001 | <0.001 |
| **PLGA NP** |  |  |  |  |  |  |  | 0.001 | 0.007 |
| **SLN** |  |  |  |  |  |  |  |  | 0.009 |
| **NLC** |  |  |  |  |  |  |  |  |  |

*10.2* *CsA biodistribution in porcine skin*

**Table S8.** P values for CsA delivery from nanoformulations in different skin layers

|  | **Stratum corneum + Viable epidermis (0-160 µm)** | | | | | | | | |
| --- | --- | --- | --- | --- | --- | --- | --- | --- | --- |
|  | **Control formulation** | **NE** | **ME** | **TPGS micelles** | **mPEG-dihexPLA micelles** | **Liposomes** | **PLGA NP** | **SLN** | **NLC** |
| **Control formulation** |  | <0.001 | <0.001 | <0.001 | <0.001 | <0.001 | <0.001 | <0.001 | <0.001 |
| **NE** |  |  | <0.001 | <0.001 | <0.001 | <0.001 | <0.001 | <0.001 | <0.001 |
| **ME** |  |  |  | <0.001 | 0.830 | <0.001 | 0.002 | 0.230 | <0.001 |
| **TPGS micelles** |  |  |  |  | <0.001 | 0.065 | <0.001 | <0.001 | <0.001 |
| **mPEG-dihexPLA micelles** |  |  |  |  |  | <0.001 | <0.001 | 0.236 | <0.001 |
| **Liposomes** |  |  |  |  |  |  | <0.001 | <0.001 | <0.001 |
| **PLGA NP** |  |  |  |  |  |  |  | <0.001 | 0.005 |
| **SLN** |  |  |  |  |  |  |  |  | <0.001 |
| **NLC** |  |  |  |  |  |  |  |  |  |
|  | **Upper dermis (160-400 µm)** | | | | | | | | |
|  | **Control formulation** | **NE** | **ME** | **TPGS micelles** | **mPEG-dihexPLA micelles** | **Liposomes** | **PLGA NP** | **SLN** | **NLC** |
| **Control formulation** |  | <0.001 | <0.001 | <0.001 | <0.001 | <0.001 | <0.001 | <0.001 | <0.001 |
| **NE** |  |  | <0.001 | <0.001 | <0.001 | <0.001 | <0.001 | <0.001 | <0.001 |
| **ME** |  |  |  |  | <0.001 | <0.001 | <0.001 | <0.001 | 0.002 |
| **TPGS micelles** |  |  |  |  | <0.001 | 0.097 | 0.153 | <0.001 | <0.001 |
| **mPEG-dihexPLA micelles** |  |  |  |  |  | <0.001 | 0.021 | 0.101 | 0.024 |
| **Liposomes** |  |  |  |  |  |  | 0.070 | <0.001 | <0.001 |
| **PLGA NP** |  |  |  |  |  |  |  | <0.001 | 0.261 |
| **SLN** |  |  |  |  |  |  |  |  | <0.001 |
| **NLC** |  |  |  |  |  |  |  |  |  |
|  | **Lower dermis (400-800 µm)** | | | | | | | | |
|  | **Control formulation** | **NE** | **ME** | **TPGS micelles** | **mPEG-dihexPLA micelles** | **Liposomes** | **PLGA NP** | **SLN** | **NLC** |
| **Control formulation** |  | <0.001 | <0.001 | <0.001 | <0.001 | 0.028 | 0.053 | <0.001 | <0.001 |
| **NE** |  |  | <0.001 | <0.001 | <0.001 | <0.001 | <0.001 | 0.012 | <0.001 |
| **ME** |  |  |  | <0.001 | 0.355 | <0.001 | 0.007 | 0.097 | 0.578 |
| **TPGS micelles** |  |  |  |  | <0.001 | 0.394 | 0.915 | <0.001 | 0.002 |
| **mPEG-dihexPLA micelles** |  |  |  |  |  | <0.001 | 0.001 | 0.315 | 0.021 |
| **Liposomes** |  |  |  |  |  |  | 0.266 | <0.001 | <0.001 |
| **PLGA NP** |  |  |  |  |  |  |  | 0.003 | 0.006 |
| **SLN** |  |  |  |  |  |  |  |  | 0.034 |
| **NLC** |  |  |  |  |  |  |  |  |  |

**Table S9.** P values for CsA delivery from different nanoformulations in individual sections from skin biodistribution studies (12 h)

|  | **Skin depth 0-40 µm** | | | | | | | | |
| --- | --- | --- | --- | --- | --- | --- | --- | --- | --- |
|  | **Control formulation** | **NE** | **ME** | **TPGS micelles** | **mPEG-dihexPLA micelles** | **Liposomes** | **PLGA NP** | **SLN** | **NLC** |
| **Control formulation** |  | <0.001 | <0.001 | <0.001 | <0.001 | <0.001 | <0.001 | <0.001 | <0.001 |
| **NE** |  |  | <0.001 | <0.001 | <0.001 | <0.001 | <0.001 | <0.001 | <0.001 |
| **ME** |  |  |  | <0.001 | 0.029 | <0.001 | <0.001 | 0.958 | <0.001 |
| **TPGS micelles** |  |  |  |  | <0.001 | 0.613 | <0.001 | <0.001 | <0.001 |
| **mPEG-dihexPLA micelles** |  |  |  |  |  | <0.001 | <0.001 | 0.047 | <0.001 |
| **Liposomes** |  |  |  |  |  |  | <0.001 | <0.001 | <0.001 |
| **PLGA NP** |  |  |  |  |  |  |  | <0.001 | 0.003 |
| **SLN** |  |  |  |  |  |  |  |  | <0.001 |
| **NLC** |  |  |  |  |  |  |  |  |  |
|  | **Skin depth 40-80 µm** | | | | | | | | |
|  | **Control formulation** | **NE** | **ME** | **TPGS micelles** | **mPEG-dihexPLA micelles** | **Liposomes** | **PLGA NP** | **SLN** | **NLC** |
| **Control formulation** |  | <0.001 | <0.001 | <0.001 | <0.001 | <0.001 | <0.001 | <0.001 | <0.001 |
| **NE** |  |  | <0.001 | <0.001 | <0.001 | <0.001 | <0.001 | <0.001 | <0.001 |
| **ME** |  |  |  | 0.008 | 0.059 | 0.003 | 0.402 | 0.042 | 0.102 |
| **TPGS micelles** |  |  |  |  | <0.001 | 0.022 | <0.001 | <0.001 | <0.001 |
| **mPEG-dihexPLA micelles** |  |  |  |  |  | <0.001 | <0.001 | 0.300 | <0.001 |
| **Liposomes** |  |  |  |  |  |  | <0.001 | <0.001 | <0.001 |
| **PLGA NP** |  |  |  |  |  |  |  | <0.010 | 0.030 |
| **SLN** |  |  |  |  |  |  |  |  | 0.004 |
| **NLC** |  |  |  |  |  |  |  |  |  |
|  | **Skin depth 80-120 µm** | | | | | | | | |
|  | **Control formulation** | **NE** | **ME** | **TPGS micelles** | **mPEG-dihexPLA micelles** | **Liposomes** | **PLGA NP** | **SLN** | **NLC** |
| **Control formulation** |  | <0.001 | <0.001 | <0.001 | <0.001 | <0.001 | <0.001 | <0.001 | <0.001 |
| **NE** |  |  | <0.001 | <0.001 | <0.001 | <0.001 | <0.001 | <0.001 | <0.001 |
| **ME** |  |  |  | <0.001 | 0.032 | <0.001 | 0.010 | 0.001 | 0.036 |
| **TPGS micelles** |  |  |  |  | <0.001 | 0.398 | <0.001 | <0.001 | <0.001 |
| **mPEG-dihexPLA micelles** |  |  |  |  |  | <0.001 | 0.001 | 0.087 | <0.001 |
| **Liposomes** |  |  |  |  |  |  | <0.001 | <0.001 | <0.001 |
| **PLGA NP** |  |  |  |  |  |  |  | <0.001 | 0.981 |
| **SLN** |  |  |  |  |  |  |  |  | <0.001 |
| **NLC** |  |  |  |  |  |  |  |  |  |
|  | **Skin depth 120-160 µm** | | | | | | | | |
|  | **Control formulation** | **NE** | **ME** | **TPGS micelles** | **mPEG-dihexPLA micelles** | **Liposomes** | **PLGA NP** | **SLN** | **NLC** |
| **Control formulation** |  | <0.001 | <0.001 | <0.001 | <0.001 | <0.001 | <0.001 | <0.001 | <0.001 |
| **NE** |  |  | <0.001 | <0.001 | <0.001 | <0.001 | <0.001 | <0.001 | <0.001 |
| **ME** |  |  |  | <0.001 | 0.735 | <0.001 | 0.010 | 0.002 | 0.003 |
| **TPGS micelles** |  |  |  |  | <0.001 | 0.302 | <0.001 | <0.001 | <0.001 |
| **mPEG-dihexPLA micelles** |  |  |  |  |  | <0.001 | 0.007 | <0.001 | 0.002 |
| **Liposomes** |  |  |  |  |  |  | <0.001 | <0.001 | <0.001 |
| **PLGA NP** |  |  |  |  |  |  |  | <0.001 | 0.709 |
| **SLN** |  |  |  |  |  |  |  |  | <0.001 |
| **NLC** |  |  |  |  |  |  |  |  |  |
|  | **Skin depth 160-200 µm** | | | | | | | | |
|  | **Control formulation** | **NE** | **ME** | **TPGS micelles** | **mPEG-dihexPLA micelles** | **Liposomes** | **PLGA NP** | **SLN** | **NLC** |
| **Control formulation** |  | <0.001 | <0.001 | <0.001 | <0.001 | <0.001 | <0.001 | <0.001 | <0.001 |
| **NE** |  |  | <0.001 | <0.001 | <0.001 | <0.001 | <0.001 | <0.001 | <0.001 |
| **ME** |  |  |  | <0.001 | 0.005 | <0.001 | <0.001 | 0.654 | <0.001 |
| **TPGS micelles** |  |  |  |  | <0.001 | 0.05 | 0.002 | <0.001 | <0.001 |
| **mPEG-dihexPLA micelles** |  |  |  |  |  | <0.001 | 0.051 | 0.138 | 0.155 |
| **Liposomes** |  |  |  |  |  |  | <0.001 | <0.001 | <0.001 |
| **PLGA NP** |  |  |  |  |  |  |  | 0.011 | 0.165 |
| **SLN** |  |  |  |  |  |  |  |  | 0.022 |
| **NLC** |  |  |  |  |  |  |  |  |  |
|  | **Skin depth 200-240 µm** | | | | | | | | |
|  | **Control formulation** | **NE** | **ME** | **TPGS micelles** | **mPEG-dihexPLA micelles** | **Liposomes** | **PLGA NP** | **SLN** | **NLC** |
| **Control formulation** |  | <0.001 | <0.001 | <0.001 | <0.001 | <0.001 | <0.001 | <0.001 | <0.001 |
| **NE** |  |  | <0.001 | <0.001 | <0.001 | <0.001 | <0.001 | <0.001 | <0.001 |
| **ME** |  |  |  | <0.001 | <0.001 | <0.001 | <0.001 | <0.001 | <0.001 |
| **TPGS micelles** |  |  |  |  | <0.001 | 0.379 | 0.139 | <0.001 | <0.001 |
| **mPEG-dihexPLA micelles** |  |  |  |  |  | <0.001 | 0.780 | 0.001 | 0.623 |
| **Liposomes** |  |  |  |  |  |  | 0.214 | <0.001 | 0.003 |
| **PLGA NP** |  |  |  |  |  |  |  | 0.005 | 0.923 |
| **SLN** |  |  |  |  |  |  |  |  | <0.001 |
| **NLC** |  |  |  |  |  |  |  |  |  |
|  | **Skin depth 240-280 µm** | | | | | | | | |
|  | **Control formulation** | **NE** | **ME** | **TPGS micelles** | **mPEG-dihexPLA micelles** | **Liposomes** | **PLGA NP** | **SLN** | **NLC** |
| **Control formulation** |  | <0.001 | <0.001 | <0.001 | <0.001 | <0.001 | <0.001 | <0.001 | <0.001 |
| **NE** |  |  | <0.001 | <0.001 | <0.001 | <0.001 | <0.001 | <0.001 | <0.001 |
| **ME** |  |  |  | <0.001 | 0.011 | <0.001 | <0.001 | 0.007 | <0.001 |
| **TPGS micelles** |  |  |  |  | 0.031 | 0.284 | 0.376 | <0.001 | 0.011 |
| **mPEG-dihexPLA micelles** |  |  |  |  |  | 0.015 | 0.185 | 0.235 | 0.173 |
| **Liposomes** |  |  |  |  |  |  | 0.189 | <0.001 | <0.001 |
| **PLGA NP** |  |  |  |  |  |  |  | 0.005 | 0.713 |
| **SLN** |  |  |  |  |  |  |  |  | <0.001 |
| **NLC** |  |  |  |  |  |  |  |  |  |
|  | **Skin depth 280-320 µm** | | | | | | | | |
|  | **Control formulation** | **NE** | **ME** | **TPGS micelles** | **mPEG-dihexPLA micelles** | **Liposomes** | **PLGA NP** | **SLN** | **NLC** |
| **Control formulation** |  | <0.001 | <0.001 | <0.001 | <0.001 | <0.001 | <0.001 | <0.001 | <0.001 |
| **NE** |  |  | <0.001 | <0.001 | <0.001 | <0.001 | <0.001 | <0.001 | <0.001 |
| **ME** |  |  |  | <0.001 | <0.001 | <0.001 | <0.001 | 0.003 | <0.001 |
| **TPGS micelles** |  |  |  |  | 0.001 | 0.002 | 0.002 | <0.001 | <0.001 |
| **mPEG-dihexPLA micelles** |  |  |  |  |  | 0.002 | 0.004 | 0.131 | 0.036 |
| **Liposomes** |  |  |  |  |  |  | 0.002 | <0.001 | <0.001 |
| **PLGA NP** |  |  |  |  |  |  |  | <0.001 | <0.001 |
| **SLN** |  |  |  |  |  |  |  |  | 0.002 |
| **NLC** |  |  |  |  |  |  |  |  |  |
|  | **Skin depth 320-360 µm** | | | | | | | | |
|  | **Control formulation** | **NE** | **ME** | **TPGS micelles** | **mPEG-dihexPLA micelles** | **Liposomes** | **PLGA NP** | **SLN** | **NLC** |
| **Control formulation** |  | <0.001 | <0.001 | 0.002 | 0.002 | 0.017 | <0.001 | <0.001 | <0.001 |
| **NE** |  |  | <0.001 | <0.001 | <0.001 | <0.001 | <0.001 | <0.001 | <0.001 |
| **ME** |  |  |  | <0.001 | 0.063 | <0.001 | <0.001 | 0.476 | 0.002 |
| **TPGS micelles** |  |  |  |  | 0.046 | 0.012 | 0.256 | 0.002 | <0.001 |
| **mPEG-dihexPLA micelles** |  |  |  |  |  | 0.012 | 0.071 | 0.132 | 0.589 |
| **Liposomes** |  |  |  |  |  |  | <0.001 | <0.001 | <0.001 |
| **PLGA NP** |  |  |  |  |  |  |  | 0.002 | <0.001 |
| **SLN** |  |  |  |  |  |  |  |  | 0.028 |
| **NLC** |  |  |  |  |  |  |  |  |  |
|  | **Skin depth 360-400 µm** | | | | | | | | |
|  | **Control formulation** | **NE** | **ME** | **TPGS micelles** | **mPEG-dihexPLA micelles** | **Liposomes** | **PLGA NP** | **SLN** | **NLC** |
| **Control formulation** |  | <0.001 | <0.001 | 0.009 | <0.001 | 0.011 | 0.054 | <0.001 | <0.001 |
| **NE** |  |  | 0.010 | <0.001 | <0.001 | <0.001 | <0.001 | <0.001 | <0.001 |
| **ME** |  |  |  | <0.001 | 0.124 | <0.001 | <0.001 | <0.001 | <0.001 |
| **TPGS micelles** |  |  |  |  | <0.001 | 0.062 | 0.290 | <0.001 | <0.001 |
| **mPEG-dihexPLA micelles** |  |  |  |  |  | <0.001 | <0.001 | <0.001 | <0.001 |
| **Liposomes** |  |  |  |  |  |  | 0.097 | <0.001 | <0.001 |
| **PLGA NP** |  |  |  |  |  |  |  | 0.002 | 0.024 |
| **SLN** |  |  |  |  |  |  |  |  | 0.021 |
| **NLC** |  |  |  |  |  |  |  |  |  |
